# A Genetic Investigation of Sex Bias in the Prevalence of Attention-Deficit/Hyperactivity Disorder

**DOI:** 10.1016/j.biopsych.2017.11.026

**Published:** 2018-06-15

**Authors:** Joanna Martin, Raymond K. Walters, Ditte Demontis, Manuel Mattheisen, S. Hong Lee, Elise Robinson, Isabell Brikell, Laura Ghirardi, Henrik Larsson, Paul Lichtenstein, Nicholas Eriksson, 23andMe Research Team, Michelle Agee, Babak Alipanahi, Adam Auton, Robert K. Bell, Katarzyna Bryc, Sarah L. Elson, Pierre Fontanillas, Nicholas A. Furlotte, David A. Hinds, Bethann S. Hromatka, Karen E. Huber, Aaron Kleinman, Nadia K. Litterman, Matthew H. McIntyre, Joanna L. Mountain, Carrie A.M. Northover, Steven J. Pitts, J. Fah Sathirapongsasuti, Olga V. Sazonova, Janie F. Shelton, Suyash Shringarpure, Chao Tian, Joyce Y. Tung, Vladimir Vacic, Catherine H. Wilson, Psychiatric Genomics Consortium: ADHD Subgroup, Özgür Albayrak, Richard J.L. Anney, Alejandro Arias Vasquez, Maria Jesús Arranz, Philip Asherson, Tobias Banaschewski, Tobias J. Banaschewski, Claiton Bau, Joseph Biederman, Preben Bo Mortensen, Anders Børglum, Jan K. Buitelaar, Miguel Casas, Alice Charach, Bru Cormand, Jennifer Crosbie, Soeren Dalsgaard, Mark J. Daly, Ditte Demontis, Astrid Dempfle, Alysa E. Doyle, Richard P. Ebstein, Josephine Elia, Stephen V. Faraone, Stephen V. Faraone, Manuel Föcker, Barbara Franke, Christine Freitag, Joel Gelernter, Michael Gill, Eugenio Grevet, Jan Haavik, Hakon Hakonarson, Ziarih Hawi, Johannes Hebebrand, Beate Herpertz-Dahlmann, Amaia Hervas, Anke Hinney, Sarah Hohmann, Peter Holmans, Mara Hutz, Abel Ickowitz, Stefan Johansson, Lindsey Kent, Sarah Kittel-Schneider, Henry Kranzler, Jonna Kuntsi, Nanda Lambregts-Rommelse, Kate Langley, Gerd Lehmkuhl, Klaus-Peter Lesch, Sandra K. Loo, Joanna Martin, James J. McGough, Sarah E. Medland, Jobst Meyer, Eric Mick, Frank Middletion, Ana Miranda, Fernando Mulas, Aisling Mulligan, Benjamin M. Neale, Stan F. Nelson, T. Trang Nguyen, Michael C. O’Donovan, Robert D. Oades, Michael J. Owen, Haukur Palmason, Josep Antoni Ramos-Quiroga, Andreas Reif, Tobias J. Renner, Luis Rhode, Marta Ribasés, Marcella Rietschel, Stephan Ripke, Olga Rivero, Herbert Roeyers, Marcel Romanos, Jasmin Romanos, Nina Roth Mota, Aribert Rothenberger, Cristina Sánchez-Mora, Russell Schachar, Helmut Schäfer, André Scherag, Benno G. Schimmelmann, Joseph Sergeant, Judith Sinzig, Susan L. Smalley, Edmund J.S. Sonuga-Barke, Hans-Christoph Steinhausen, Patrick F. Sullivan, Anita Thapar, Margaret Thompsom, Alexandre Todorov, Irwin Waldman, Susanne Walitza, Raymond Walters, Yufeng Wang, Andreas Warnke, Nigel Williams, Stephanie H. Witt, Li Yang, Tetyana Zayats, Yanli Zhang-James, iPSYCH–Broad ADHD Workgroup, Esben Agerbo, Thomas Damm Als, Marie Bækved-Hansen, Rich Belliveau, Anders D. Børglum, Jonas Bybjerg-Grauholm, Felecia Cerrato, Kimberly Chambert, Claire Churchhouse, Søren Dalsgaard, Mark J. Daly, Ditte Demontis, Ashley Dumont, Jacqueline Goldstein, Jakob Grove, Christine S. Hansen, Mads Engel Hauberg, Mads V. Hollegaard, David M. Hougaard, Daniel P. Howrigan, Hailiang Huang, Julian Maller, Alicia R. Martin, Joanna Martin, Manuel Mattheisen, Jennifer Moran, Ole Mors, Preben Bo Mortensen, Benjamin M. Neale, Merete Nordentoft, Jonatan Pallesen, Duncan S. Palmer, Carsten Bøcker Pedersen, Marianne Giørtz Pedersen, Timothy Poterba, Jesper Buchhave Poulsen, Stephan Ripke, Elise B. Robinson, F. Kyle Satterstrom, Christine Stevens, Patrick Turley, Raymond K. Walters, Thomas Werge, Thomas Werge, Preben Bo Mortensen, Marianne Giørtz Pedersen, Ole Mors, Merete Nordentoft, David M. Hougaard, Jonas Bybjerg-Grauholm, Naomi R. Wray, Barbara Franke, Stephen V. Faraone, Michael C. O’Donovan, Anita Thapar, Anders D. Børglum, Benjamin M. Neale

**Affiliations:** a Department of Medical Epidemiology & Biostatistics, Karolinska Institutet, Stockholm, Sweden; b Centre for Psychiatry Research, Department of Clinical Neuroscience, Karolinska Institutet, Stockholm, Sweden; c Stockholm Health Care Services, Stockholm County Council, Stockholm, Sweden; d School of Medical Sciences, Örebro University, Örebro, Sweden; e Stanley Center for Psychiatric Research, Broad Institute, Cambridge, Massachusetts; f Analytic and Translational Genetics Unit, Massachusetts General Hospital, Boston, Massachusetts; g 23andMe Inc., Mountain View, California; h Departments of Psychiatry and of Neuroscience and Physiology, SUNY Upstate Medical University, Syracuse, New York; i MRC Centre for Neuropsychiatric Genetics and Genomics, Cardiff University, Cardiff, United Kingdom; j Lundbeck Foundation Initiative for Integrative Psychiatric Research [iPSYCH], Aarhus, Roskilde, Denmark; k Centre for Integrative Sequencing [iSEQ], Aarhus University, Aarhus, Roskilde, Denmark; l Department of Biomedicine–Human Genetics, Aarhus University, Aarhus, Roskilde, Denmark; m National Centre for Register-Based Research, Aarhus University, Aarhus, Roskilde, Denmark; n Centre for Integrated Register-Based Research, Aarhus University, Aarhus, Roskilde, Denmark; o Institute of Biological Psychiatry, MHC Sct. Hans, Mental Health Services Copenhagen, Roskilde, Denmark; p Department of Clinical Medicine, University of Copenhagen, Copenhagen, Denmark; q Mental Health Services in the Capital Region of Denmark, Mental Health Center Copenhagen, University of Copenhagen, Copenhagen, Denmark; r Center for Neonatal Screening, Department for Congenital Disorders, Statens Serum Institut, Copenhagen, Denmark; s Psychosis Research Unit, Aarhus University Hospital, Risskov, Denmark; t Queensland Brain Institute, University of Queensland, Brisbane, Queensland, Australia; u School of Environmental and Rural Science, University of New England, Armidale, New South Wales, Australia; v Centre for Population Health Research, School of Health Sciences and Sansom Institute of Health Research, University of South Australia, Adelaide, Australia; w Departments of Human Genetics and Psychiatry, Donders Institute for Brain, Cognition and Behaviour, Radboud University Medical Center, Nijmegen, The Netherlands; x K.G. Jebsen Centre for Research on Neuropsychiatric Disorders, University of Bergen, Bergen, Norway

**Keywords:** ADHD, Epidemiology, GWAS, Neurodevelopmental disorders, Polygenic risk score analysis, Sex bias

## Abstract

**Background:**

Attention-deficit/hyperactivity disorder (ADHD) shows substantial heritability and is two to seven times more common in male individuals than in female individuals. We examined two putative genetic mechanisms underlying this sex bias: sex-specific heterogeneity and higher burden of risk in female cases.

**Methods:**

We analyzed genome-wide autosomal common variants from the Psychiatric Genomics Consortium and iPSYCH Project (*n* = 20,183 cases, *n* = 35,191 controls) and Swedish population register data (*n* = 77,905 cases, *n* = 1,874,637 population controls).

**Results:**

Genetic correlation analyses using two methods suggested near complete sharing of common variant effects across sexes, with *r*_g_ estimates close to 1. Analyses of population data, however, indicated that female individuals with ADHD may be at especially high risk for certain comorbid developmental conditions (i.e., autism spectrum disorder and congenital malformations), potentially indicating some clinical and etiological heterogeneity. Polygenic risk score analysis did not support a higher burden of ADHD common risk variants in female cases (odds ratio [confidence interval] = 1.02 [0.98–1.06], *p* = .28). In contrast, epidemiological sibling analyses revealed that the siblings of female individuals with ADHD are at higher familial risk for ADHD than the siblings of affected male individuals (odds ratio [confidence interval] = 1.14 [1.11–1.18], *p* = 1.5E-15).

**Conclusions:**

Overall, this study supports a greater familial burden of risk in female individuals with ADHD and some clinical and etiological heterogeneity, based on epidemiological analyses. However, molecular genetic analyses suggest that autosomal common variants largely do not explain the sex bias in ADHD prevalence.

SEE COMMENTARY ON PAGE e55

Attention-deficit/hyperactivity disorder (ADHD) is a common (∼5% childhood prevalence), highly heritable (70%–80%) neurodevelopmental disorder 1, 2. Recent genome-wide association studies (GWASs) implicate thousands of genetic risk variants across the allele frequency spectrum 3, 4, 5, 6, 7. The first robust genome-wide significant single nucleotide polymorphisms (SNPs) were recently identified in a GWAS meta-analysis of 20,183 ADHD cases and 35,191 controls/pseudocontrols (7). While such efforts are beginning to shed light on ADHD biology, secondary genome-wide analyses can address important issues regarding the etiological and clinical heterogeneity of ADHD.

In children, boys show a two to seven times higher ADHD diagnosis rate than girls 1, 8. The male excess is more pronounced in individuals ascertained from clinics than from the community, and this difference attenuates during adulthood (2). The reasons for the difference in childhood prevalence are unclear. Here, we present a series of analyses aimed at elucidating the basis for this difference.

### Sex-Specific Heterogeneity

One possibility is that female ADHD is qualitatively different from male ADHD. Although the majority of twin studies have not detected any quantitative or qualitative sex differences in ADHD heritability 9, 10, 11, 12, this does not necessarily imply that the same genetic variants are involved in ADHD etiology in both sexes. If ADHD in clinically diagnosed male individuals is distinct from ADHD in diagnosed female individuals, this could yield differences in prevalence. Sex-based genetic heterogeneity in common variants has been shown for several complex human traits (e.g., blood pressure, waist-hip ratio) 13, 14. Here, we assessed the genome-wide autosomal genetic correlation of ADHD in male and female individuals to determine whether genetic heterogeneity from common variation contributes to the observed biased prevalence.

The absence of extensive sequencing data currently precludes analogous analyses of rare genetic variants. Instead, to evaluate whether such variants play differential roles in male and female individuals with ADHD, we used risk for comorbid brain-related developmental disorders (i.e., autism spectrum disorder [ASD], intellectual disability [ID], epilepsy, motor developmental delay) and rare syndromic phenotypes (i.e., congenital malformations, syndromes related to chromosomal abnormalities) as proxies for possible presence of de novo or rare segregating alleles. Rare, highly deleterious (including noninherited) genetic variation has been implicated in such phenotypes 15, 16, 17, 18, 19, 20, 21, 22, 23, 24, 25. Indeed, comorbid ID has been associated with an increased likelihood that an individual with ADHD is a carrier of a large, rare copy number variant (CNV) (26). It has also long been known that rare genetic syndromes (e.g., fragile X syndrome, velocardiofacial syndrome) are associated with ADHD 27, 28. Evidence for an increase in comorbid conditions in female individuals with ADHD, when compared with affected male individuals, would imply a more severe syndromal phenotypic presentation of ADHD in a higher proportion of female individuals. Such more complex presentations are arguably more likely to be linked to deleterious rare mutations. A higher rate of these comorbidities in female individuals would also be consistent with clinical heterogeneity, which may pertain to the observed prevalence differences.

### Female Protective Effect

Aside from heterogeneity, prevalence differences may be caused by a female protective effect, whereby female individuals are resilient to developing ADHD and thus require a higher burden of genetic liability to develop it. Three family studies have observed indirect evidence for this hypothesis in the form of increased risk of ADHD in first-degree relatives of affected female individuals compared with affected male individuals, suggesting that families with an affected female individual may have a higher burden of genetic risk 29, 30, 31. Not all studies report an increase in the recurrence rate of ADHD in relatives of female probands, however 32, 33. Two molecular genetic studies tested this hypothesis more directly using ADHD GWAS discovery data to calculate the burden of common risk alleles, as estimated by polygenic risk scores (PRSs), in independent samples. In both studies, female children with ADHD-related phenotypes had higher scores for ADHD than affected male individuals 3, 34. Although these preliminary studies are consistent with the family studies mentioned above, they were based on small discovery studies. Additional tests using large GWAS datasets are needed to test whether there is an increased burden of common genetic risk variants in female individuals with ADHD.

We present a series of analyses to test the qualitative and quantitative difference hypotheses for the biased sex prevalence in ADHD. First, autosomal common variant data were used to estimate the genetic correlation of ADHD in male and female individuals. We then used population register data to examine whether female individuals with ADHD are at an increased risk for comorbid developmental conditions compared with affected male individuals. Next, genome-wide autosomal SNP data were used to test whether female individuals diagnosed with ADHD carry a higher burden of common risk variants than affected male individuals. Finally, using register data, we tested whether relatives of female individuals with ADHD are at an increased risk for ADHD compared with relatives of diagnosed male individuals.

## Methods and Materials

### Genetic Data

Data for ADHD cases and controls were available from the Psychiatric Genomics Consortium (PGC) and the Lundbeck Foundation Initiative for Integrative Psychiatric Research (iPSYCH). See GWAS publication for full details of quality control, imputation, and principal components analysis (7) and the Supplement for a more detailed description of all methods.

The iPSYCH samples were genotyped, processed, and analyzed in 23 separate waves. See Supplemental Table S1 for sex-stratified sample sizes for each PGC study and iPSYCH wave. The total sample size after all quality control was *n* = 20,183 cases (25% female) and *n* = 35,191 pseudocontrols/population controls (38% female). Analyses that were restricted to European-only samples consisted of 19,099 cases and 34,194 controls. ADHD GWAS summary statistics were also available from research participants of the personal genetics company 23andMe Inc. (*n* = 5857 self-reported ADHD cases, *n* = 70,393 controls) (Mountain View, CA).

### GWAS Analyses

Sex-specific logistic regression GWAS analyses of imputed autosomal data were performed in each PGC study and iPSYCH wave separately. Results were filtered for each study/wave based on minor allele frequency, imputation quality, call rate, and expected minor allele frequency in cases. Sex-specific GWAS results for samples of European ancestry were meta-analyzed and also filtered based on sample size and presence of each SNP in both sets of results. This yielded results for *n* = 7,531,543 common variants (hereafter PGC + iPSYCH). GWAS summary statistics can be downloaded at https://www.med.unc.edu/pgc/results-and-downloads.

### Testing for Sex-Specific Heterogeneity

Bivariate linkage disequilibrium (LD) score regression (LDSC) 35, 36 analyses were run on the sex-specific meta-analyzed summary statistics. The primary analyses (with greatest power) are those for the full PGC + iPSYCH sample; we also examined estimates in the PGC and iPSYCH samples separately using LDSC and a second method, genomic relatedness matrix restricted maximum likelihood (GREML), using genome-wide complex trait analysis (37). Because of strict restrictions on access to individual genotypes, bivariate GREML analyses (38) were performed separately in PGC and iPSYCH. Analyses were restricted to European-only samples. Sex-specific heritability was estimated using univariate models (see Supplement for detailed methods).

### Testing the Female Protective Effect Hypothesis

A leave-one-study/wave-out approach was used to maximize power and maintain independent target and discovery samples for PRS analyses using the standard approach 39, 40. GWAS results from 23andMe were also included in discovery meta-analyses. A PRS was calculated for each individual in each target sample (European-only samples) by scoring the number of alleles weighted by log(odds ratio [OR]) across the set of clumped, meta-analyzed SNPs in PLINK version 1.9 (see Supplemental Table S2). PRSs were standardized using *z*-score transformations; ORs can be interpreted as increase in risk of the outcome per standard deviation in PRS. Logistic regression analyses including principal components were used to test for association of PRS with sex in cases (female individuals were coded as 1). Results of these leave-one-study/wave-out analyses were meta-analyzed.

### Epidemiological Analyses

Analyses of Swedish register data were based on all individuals of known parents born in Sweden between 1987 and 2006, living in Sweden at least until 12 years of age (*n* = 77,905 ADHD cases and *n* = 1,874,637 population controls). See Supplement for details of the sample and analyses.

### Testing for Sex-Specific Heterogeneity

We assessed sex-specific differences in association between ADHD and the following categories of developmental disorders/syndromes: ID, ASD, developmental coordination disorder, epilepsy, congenital malformations, and chromosomal abnormalities (see Supplemental Table S3 for details, including International Classification of Diseases codes). Generalized estimating equations were used to test for the effect of an ADHD-by-sex interaction term on each outcome, while accounting for related samples.

### Testing the Female Protective Effect Hypothesis

We estimated whether risk of ADHD in siblings of female individuals with ADHD was higher than that in siblings of affected male individuals after adjusting for sex of the comparison sibling. Analyses stratified by sex of the comparison sibling are also presented. Analyses were restricted to pairs of full siblings where at least one child had an ADHD diagnosis (*n* = 71,691 observations, including *n* = 21,784 unique index individuals).

## Results

### Sex-Specific Heterogeneity

#### Genetic Correlation

**Figure 1:**
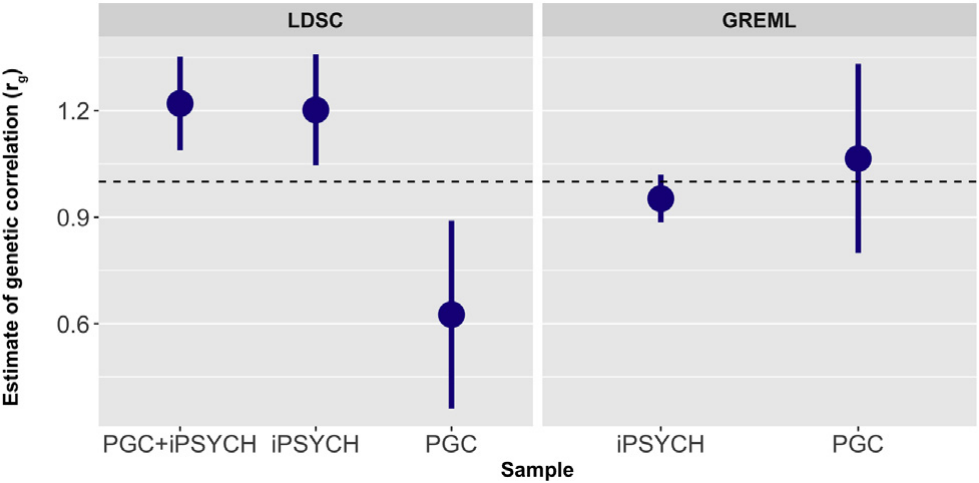
Genetic correlation estimates for attention-deficit/hyperactivity disorder in male and female individuals obtained from genomic relatedness matrix restricted maximum likelihood (GREML) and linkage disequilibrium score regression (LDSC) for the Lundbeck Foundation Initiative for Integrative Psychiatric Research (iPSYCH), Psychiatric Genomics Consortium (PGC), and combined PGC + iPSYCH datasets. Because of strict restrictions on raw individual genotype access and transfer, GREML analyses could only be performed separately in the PGC and iPSYCH samples. Bars display standard errors. The horizontal dashed line indicates a genetic correlation of 1. The estimator was left unconstrained for these analyses to allow for an unbiased assessment of the standard errors of the estimates; as such, some of the estimates exceed 1.

Figure 1 displays genetic correlation (*r*_g_) results for male and female ADHD from bivariate analyses using GREML and LDSC (see Supplemental Table S4 for exact estimates). The LDSC *r*_g_ estimate in the full dataset (PGC + iPSYCH) was near 1. Similar results were found for bivariate GREML analyses in both iPSYCH and PGC and for the LDSC analyses in the iPSYCH dataset. The LDSC estimate in the PGC dataset was lower, but large standard errors were seen in this dataset for both methods.

Additional cross-dataset and cross-sex LDSC genetic correlation estimates were used to assess the extent of heterogeneity across PGC and iPSYCH (see Supplemental Figure S1 and Table S5). The genetic correlation between these subsamples was not significantly different from 1 [*r*_g_ (SE) = 1.13 (0.22)]. Cross-dataset *r*_g_ values for PGC male individuals with iPSYCH male and female individuals were also not significantly different from 1. Estimates for PGC female individuals with iPSYCH female individuals were lower (significantly different from 0 and 1), although this is likely related to the small sample size of the PGC female individuals (*n* = 1067 cases and *n* = 5178 controls).

**Figure 2:**
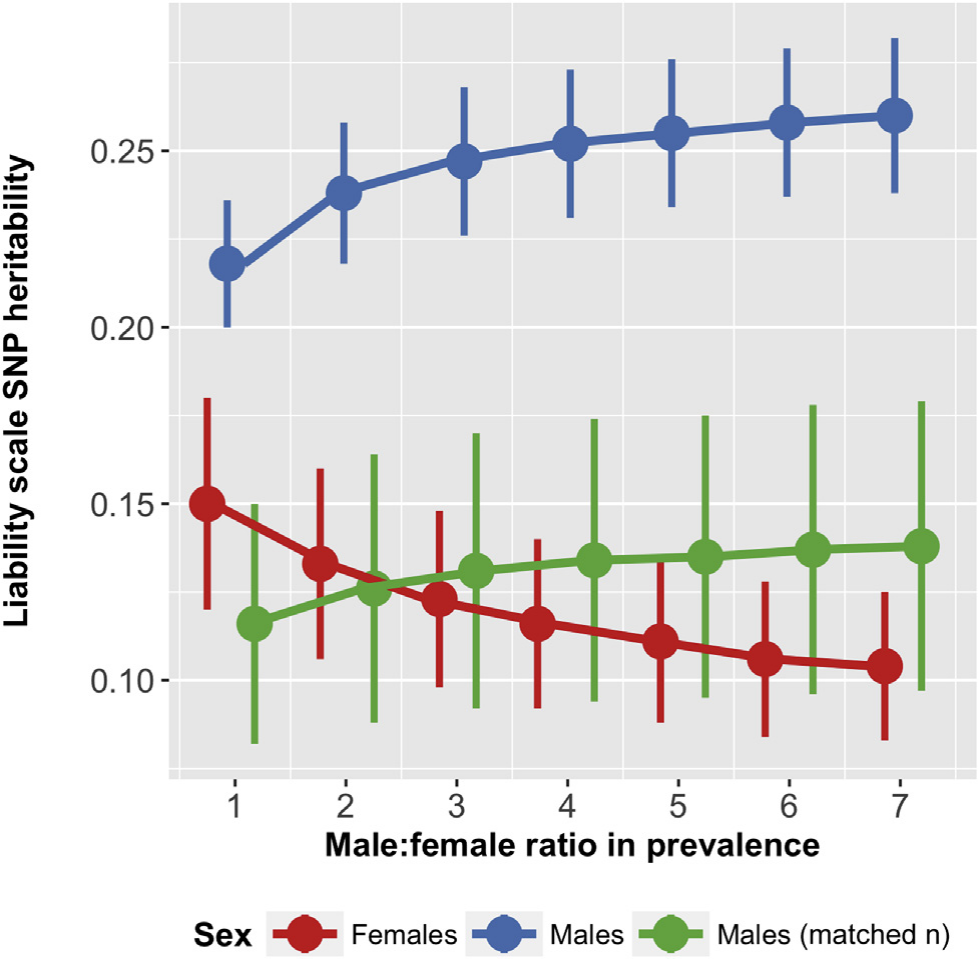
Sex-specific single nucleotide polymorphism (SNP) heritability estimates for attention-deficit/hyperactivity disorder, varying the assumed population prevalence based on different male:female ratios (ranging from 1:1 to 7:1). Estimates are presented for the total available sample of male individuals as well as for a downsampled set of male cases and controls to match the available sample size in female individuals.

SNP-h^2^ was estimated at 0.123 (SE = 0.025) in female individuals and 0.247 (SE = 0.021) in male individuals (Supplemental Figure S2 and Table S4). Downsampling male cases and controls randomly to match the female sample size and case-control ratio showed more similar SNP-h^2^ estimates (see Supplemental Figure S2 and Table S6). Results varying the relative population prevalence assumed are shown in Supplemental Table S7. Figure 2 summarizes these results, illustrating the impact of the assumed male:female ratio (which affects assumed sex-specific population prevalence rates) and sample size on SNP-h^2^ estimates; SNP-h^2^ estimates increased in male individuals and decreased in female individuals as the ratio was increased, and downsampling male cases and controls gave similar SNP-h^2^ estimates in both sexes.

#### Genome-Wide Association Studies

**Figure 3:**
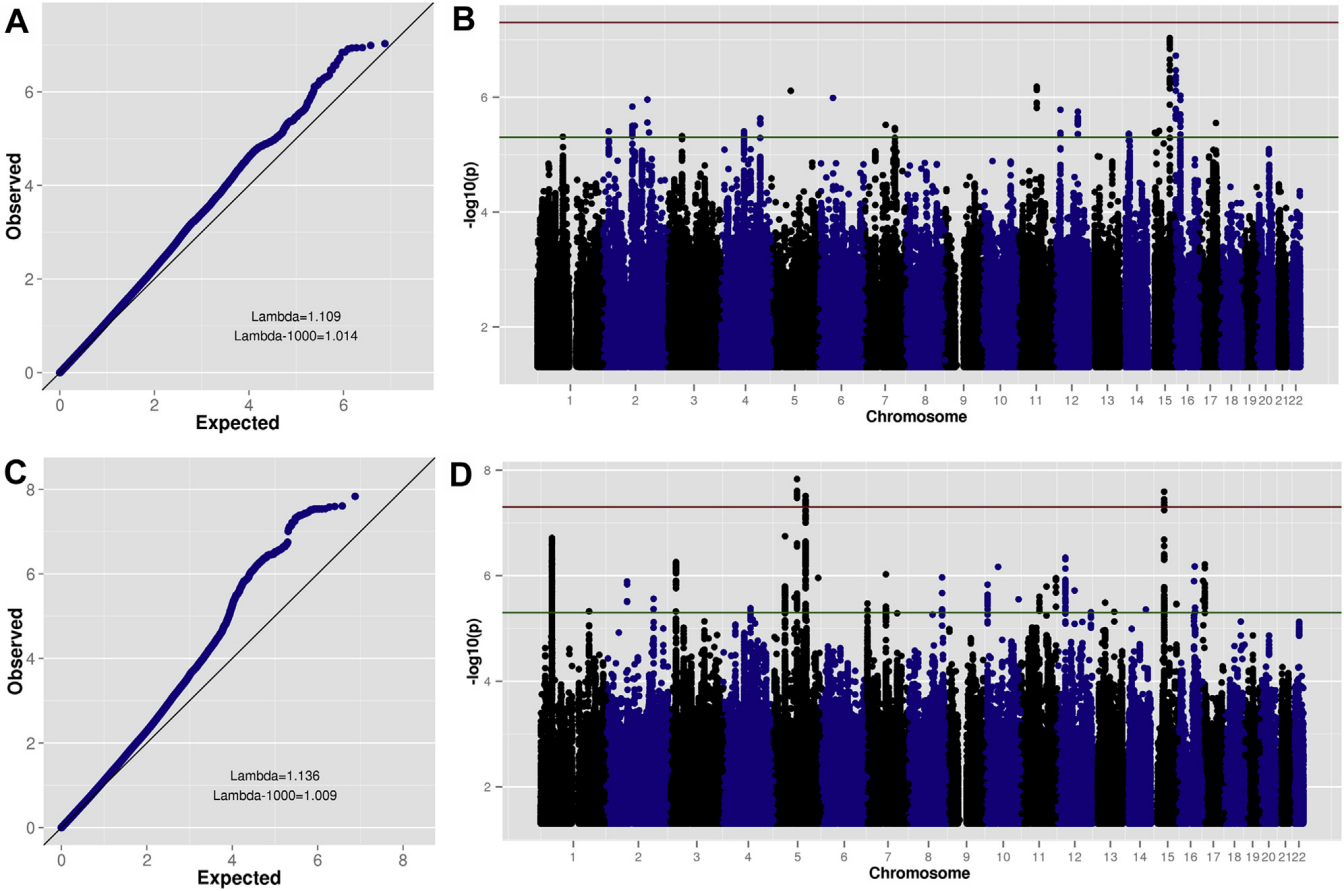
Quantile–quantile and Manhattan plots for sex-specific genome-wide association meta-analyses. **(A)** Female case-control analysis quantile–quantile plot. **(B)** Female case-control analysis Manhattan plot. **(C)** Male case-control analysis quantile–quantile plot. **(D)** Male case-control analysis Manhattan plot. In **(B)** and **(D)**, the horizontal red (upper) line indicates genome-wide significance (*p* < 5E-8) and the horizontal green (lower) line indicates suggestive subthreshold signals (*p* < 5E-6).

Sex-specific quantile–quantile and Manhattan plots are shown in Figure 3. Three loci were genome-wide significant in the male-only GWAS (*n* = 14,154 cases, *n* = 17,948 controls). No SNPs surpassed the genome-wide significance threshold in the female-only GWAS (*n* = 4945 cases, *n* = 16,246 controls). (See Supplemental Table S8 for top 10 LD-independent SNPs for each GWAS, annotated with the nearest gene.)

Several secondary analyses support the high genetic correlation, suggesting that there is little or no difference in the ADHD results for male and female individuals. First, no genome-wide significant heterogeneity is observed when meta-analyzing the sex-specific GWAS results (Supplemental Figure S3). Second, a GWAS of sex-by-genotype interactions for ADHD identifies no variants with differential effects by sex, nor does it show any deviation from the null distribution of test statistics genome-wide (Supplemental Figure S4). Similarly, GWAS results for ADHD in the combined sample with or without including sex as a covariate are nearly perfectly correlated, with a low standard error (*r*_g_ = .97, SE = .007). Narrowing the focus to only ADHD cases also finds no genome-wide significant differences between male and female cases (Supplemental Figure S5). Although some genome-wide inflation is observed for this final analysis in the iPSYCH sample, it is not replicated in the PGC data and appears to be attributable to one locus driven by a single low-frequency genotyped SNP (minor allele frequency = 0.02). Investigation of this locus shows no support for differences between male and female cases in neighboring genotyped SNPs, suggesting that the signal is likely a technical artifact (Supplemental Figure S6).

#### Epidemiological Analyses

We examined the association between having an ADHD diagnosis and risk of having a comorbid developmental phenotype using Swedish register data. We were interested in whether there is an interaction between sex and ADHD as it pertains to these comorbidities. Supplemental Table S9 displays the frequency of the disorder categories examined and the proportion of individuals affected overall and split by ADHD case status and sex. The male:female ratio for ADHD was 2:1. ADHD cases were at higher risk for all diagnostic categories, as compared with sex-matched controls (Table 1). Significant ADHD-by-sex interactions were observed for ASD and congenital malformations, suggesting that although in the context of ADHD both sexes are at increased risk for these comorbid problems, the increase in risk is even higher in female individuals compared with controls. A nominally significant association was observed for the interaction term for ID, which did not survive correction for multiple testing (Bonferroni correction for six independent tests: *p*-value threshold = .0083). Secondary analyses of severity of ID (where information was available) indicated that this weak association signal came from mild ID (IQ = 50–70), not moderate (IQ = 35–49) or severe/profound (IQ < 35) ID (Supplemental Table S10). Interaction terms were nonsignificant for epilepsy, developmental coordination disorder, and chromosomal abnormalities.

**Table 1:** Results of Logistic Regression Analyses of ADHD Case Status on Comorbid Developmental Conditions, Stratified by Sex, in the Swedish Population Sample (Total *N* = 1,952,542)

Outcome	Sex	% of ADHD Cases With Outcome	Sex-Specific Association				ADHD by Sex Interaction			
			OR	LCI	UCI	*p*	OR	LCI	UCI	*p*
ASD	Male	14.82	18.91	18.33	19.50	< 2.2E-308^a^	1.52	1.44	1.61	2.9E-50^a^
	Female	12.04	28.68	27.38	30.04	< 2.2E-308^a^				
DCD	Male	1.86	17.90	16.38	19.57	< 2.2E-308^a^	0.97	0.82	1.14	.71
	Female	1.08	17.61	15.25	20.33	< 2.2E-308^a^				
ID	Male	5.30	9.82	9.38	10.28	< 2.2E-308^a^	1.11	1.03	1.20	.0090
	Female	4.89	10.89	10.23	11.60	< 2.2E-308^a^				
Epilepsy	Male	2.57	3.27	3.09	3.47	< 2.2E-308^a^	1.08	0.99	1.19	.099
	Female	2.85	3.55	3.29	3.82	6.6E-238^a^				
Congenital Malformations	Male	8.20	1.40	1.36	1.45	1.8E-109^a^	1.11	1.05	1.17	2.5E-04^a^
	Female	6.46	1.54	1.47	1.62	8.9E-72^a^				
Chromosomal Abnormalities	Male	0.60	2.96	2.63	3.34	2.7E-70^a^	0.84	0.68	1.03	.096
	Female	0.52	2.48	2.07	2.96	9.5E-24^a^				

Sex is coded as 0 for male and 1 for female. Birth year is included as a covariate.

ADHD, attention-deficit/hyperactivity disorder; ASD, autism spectrum disorder; DCD, developmental coordination disorder; ID, intellectual disability; LCI, lower confidence interval; OR, odds ratio; UCI, upper confidence interval.

a Estimated *p* value below a Bonferroni-adjusted threshold for multiple testing (*p* < .0083).

### Female Protective Effect Hypothesis

#### PRS Analysis

**Figure 4:**
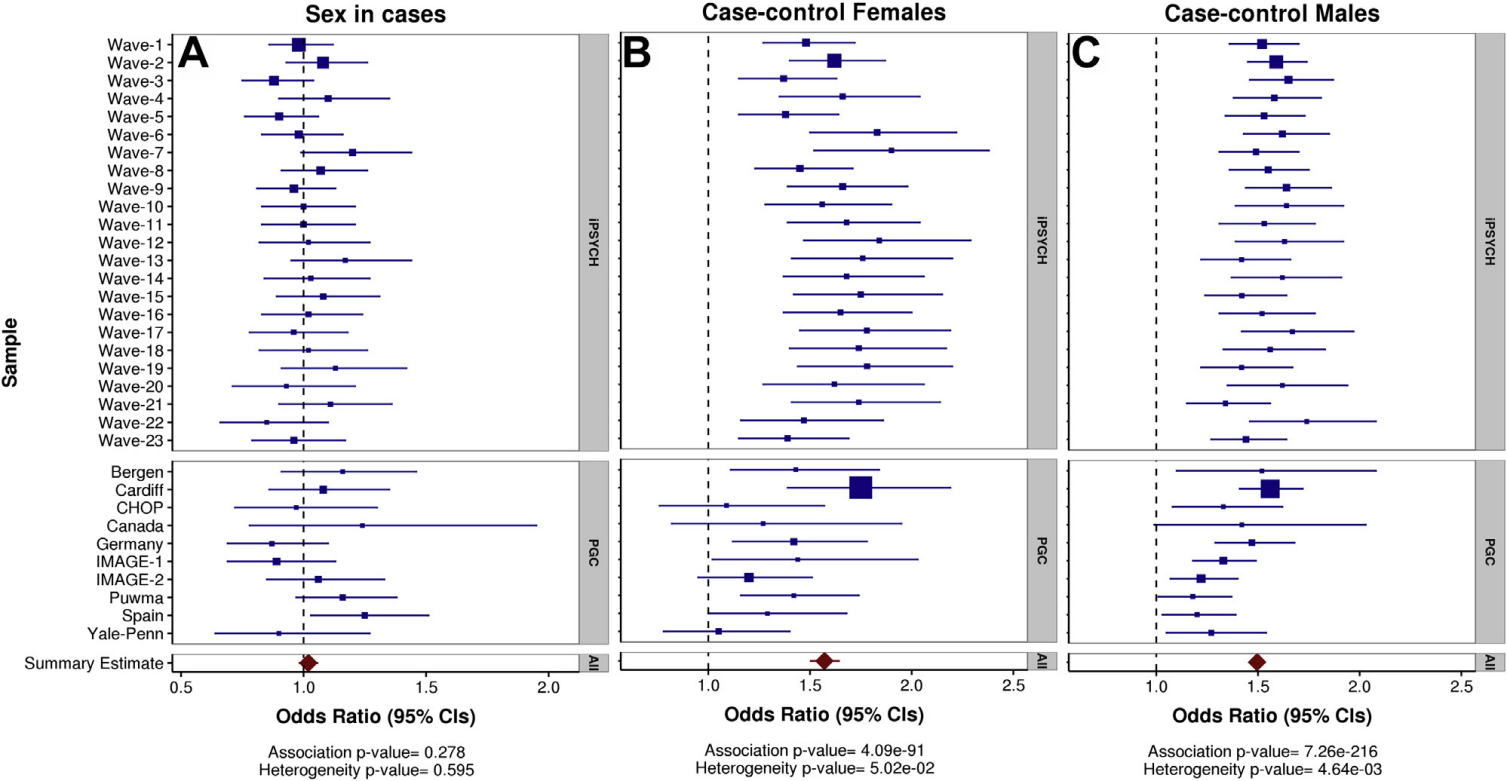
Forest plots of meta-analysis results for logistic regression analyses of attention-deficit/hyperactivity disorder polygenic risk scores with case sex as the outcome **(A)**, case-control status in female individuals **(B)**, and case-control status in male individuals **(C)**. Box sizes reflect sample sizes. Refer to Table 1 for exact sample sizes. CIs, confidence intervals; iPSYCH, Lundbeck Foundation Initiative for Integrative Psychiatric Research; PGC, Psychiatric Genomics Consortium.

Results of meta-analyses of each leave-one-study/wave-out regression analysis for ADHD PRS are shown in Figure 4. There was no association of ADHD PRS with sex in cases (OR [confidence interval (CI)] = 1.02 [0.98–1.06], *p* = .28, mean *R*^2^ [SE] = .0019 [.00039]). Sensitivity tests were run excluding the data from 23andMe and non-European ancestry individuals and then also not including sex as a covariate in the discovery GWAS analyses; results remained similar (Supplemental Figure S7). There was no association of ADHD PRS with sex in controls (OR [CI] = 0.99 [0.96–1.01], *p* = .23, mean *R*^2^ [SE] = .0011 [.00024]) (Supplemental Figure S8). Mothers and fathers (in parent-offspring trio design studies) also did not differ in ADHD PRS (Supplemental Table S11).

There was a clear association of ADHD PRS with ADHD case status, with similar differences in PRS between cases and controls observed for female individuals (OR [CI] = 1.57 [1.50–1.64], *p* = 4.1xE-91, mean *R*^2^ [SE] = .039 [.0034]) and male individuals (OR [CI] = 1.50 [1.46–1.53], *p* = 7.3xE-216, mean *R*^2^ [SE] = .032 [.0024]). In this analysis, several individual PGC studies did not show a significant association with case-control status in female individuals; this is likely due to low power (e.g., *n* = 27 female case/pseudocontrol pairs in the Canadian study).

#### Epidemiological Analysis

To test for evidence of increased risk for ADHD in siblings of female individuals with ADHD, we used Swedish population data to select sibling pairs where at least one child had ADHD. The results showed that siblings of female individuals diagnosed with ADHD are at higher risk for ADHD than siblings of affected male individuals (OR [CI] = 1.14 [1.11–1.18], *p* = 1.5E-15); results stratified by comparison sibling sex were similar for male individuals (OR [CI] = 1.18 [1.13–1.23], *p* = 1.8E-13) and female individuals (OR [CI] = 1.09 [1.01–1.16], *p* = .017). Covarying for presence of ASD, congenital malformations, and ID in the proband did not affect the overall results (OR [CI] = 1.15 [1.11–1.19], *p* = 7.3E-16).

## Discussion

We tested two specific hypotheses for the male bias in ADHD: first, that sex-specific genetic heterogeneity may affect prevalence rates via clinical heterogeneity in ADHD diagnosis by sex, and second, that female individuals affected with ADHD may carry an increased burden of genetic risk variants compared with affected male individuals. We analyzed autosomal common variant data from the largest available ADHD case-control GWAS sample (*n* = 55,374 individuals) and Swedish population register data (*n* = 1,952,542 individuals). We demonstrated a high level of genome-wide autosomal genetic correlation for ADHD across sex and found no increase of polygenic burden in affected female individuals compared with affected male individuals. However, in register-based data, we observed that siblings of female individuals with ADHD are at increased risk for ADHD compared with siblings of affected male individuals. The results also suggested that female individuals diagnosed with ADHD may be at an especially high risk for certain comorbid developmental conditions.

The observed high SNP-based genetic correlation (consistent across two methods) suggests that largely the same common autosomal risk variants are involved in ADHD for both sexes. While sex-specific heterogeneity from common autosomal variants seems unlikely based on our results, we cannot rule out the possibility that heterogeneous effects exist for rare or nonautosomal variation or that with increased sample sizes, weaker effects of common variant heterogeneity could be detected.

Indeed, the epidemiological analyses of Swedish population data suggest some degree of clinical and/or etiological heterogeneity. ADHD was associated with comorbid diagnoses of developmental conditions in both sexes. The strength of association was greater in female individuals for ASD, congenital malformations, and (to a lesser extent) mild ID, but it was similar for both sexes for motor problems, epilepsy, and chromosomal abnormalities. There are several possible explanations for these findings.

First, female individuals with ADHD may have a higher than expected risk of comorbid severe conditions and so may have a higher level of clinical heterogeneity as compared with male cases. This could indirectly point to a greater role of rare deleterious variants in female individuals with ADHD because such variants are strongly implicated in some of the comorbid conditions assessed 15, 16, 17, 18, 19, 20, 21, 22, 23, 24, 25 and this effect has been seen in other neurodevelopmental disorders 15, 16, 17, 18, 19. However, this needs to be tested directly because common variants also play an important role in such complex disorders 41, 42, and little is currently known about the contribution of rare variants to ADHD.

An alternative explanation for the results is that ascertainment and diagnostic biases, where female individuals are more likely to be diagnosed with ADHD if they have a more severe phenotypic presentation, could be involved. Individuals who receive one diagnosis become the focus of clinical attention and are more likely to receive subsequent diagnoses, whereas individuals with less complex phenotypes might not come to clinical attention. If female individuals are routinely underdiagnosed with ADHD or other neurodevelopmental disorders, this issue may disproportionately affect ascertainment of female cases, leading to the observed pattern of results. Other possible sources of bias include typical exclusion criteria for diagnosing ADHD (e.g., ID, ASD) and the possibility of an inflated false positive rate of diagnoses due to diagnostic uncertainty and change over time. We limited the impact of the latter by considering diagnoses only in individuals with at least two reported diagnoses. Although the results are consistent with increased etiological heterogeneity in female individuals with ADHD, studies of rare variation are required to rule out these alternative explanations.

Epidemiological analyses also showed that siblings of female individuals diagnosed with ADHD were at higher risk for being diagnosed with ADHD than siblings of diagnosed male individuals. This confirms results from previous family studies 29, 30, 31 indirectly supporting the hypothesis that female individuals require a greater burden of genetic risk to manifest ADHD. Although these analyses do not distinguish between genetic and shared environmental effects, twin studies have consistently demonstrated high heritability of ADHD with typically nonsignificant contributions from shared environmental factors (2), suggesting that this effect is most likely to be genetic in origin. However, the effect sizes were not large (OR = 1.09–1.18), suggesting that any increased burden of inherited genetic variation might be only a small contribution to the sex bias in ADHD prevalence. These results could also occur if clinicians had a higher threshold for diagnosing ADHD in female individuals or were more likely to diagnose it if accompanied by a comorbid disorder. However, the results did not attenuate when comorbid conditions were accounted for. Alternatively, sex-specific ascertainment biases could inflate estimated risk of ADHD in siblings of female individuals. Although female cases were more likely to be ascertained if they had an already diagnosed brother (64.6%) than vice versa, the sex-stratified results indicate that such biases cannot fully explain our results. A limitation of the analyses was restricting the sample to full siblings, which may have led to a conservative estimate of the effect size and the possibility that the results might not generalize to families with one child or half-siblings.

Contrary to smaller studies 3, 34, we did not find an enrichment of polygenic burden from common variants in female individuals compared with male individuals with ADHD. Consistently, analyses in ASD have not found an increased burden from common variants in affected female individuals 43, 44, in contrast to rare variant studies 15, 16, 17, 18, 19.

One possibility is that a higher degree of genetic heterogeneity within female individuals masked any differences in PRS burden by sex in the current study. Common and rare variants may contribute additively to ADHD risk, with non-CNV carrier cases having lower ADHD PRSs than cases with large rare CNVs (45). Thus, if female cases are more likely to have a complex syndromic phenotype (as suggested by the register-based analyses), and given that such phenotypes are more likely to be associated with rare variants 15, 16, 17, 18, 19, 20, 21, 22, 23, 24, 25, this subgroup of female individuals could have on average lower PRSs than male individuals with ADHD. On the other hand, affected female individuals with a less severe phenotypic presentation, who are not carriers of such rare variants, could have higher PRSs than affected male individuals. If this were the case, any overall differences in PRS between the sexes could be obscured. Although the variance in ADHD PRS did not appear to differ between male and female cases (*p* = .31; see Supplement), limited power of this analysis prevents us from conclusively ruling out this possibility.

Although the focus of this paper is on possible genetic sources of influence on ADHD sex bias, other factors, such as ascertainment and diagnostic biases, may play an important role. Female individuals are more likely to be diagnosed with the predominantly inattentive subtype of ADHD, whereas male individuals are more likely to be diagnosed with the combined subtype of ADHD and present with hyperactive-impulsive and disruptive behavioral problems 46, 47, 48, 49. Relative prevalence rates also vary by diagnostic instrument used and case ascertainment. For example, the ratio of male:female cases in the Swedish population was 2:1, somewhat lower than that in the iPSYCH Danish population (2.8:1) and PGC clinical data (3.5:1). Individuals with moderate to severe ID (IQ < 50) were excluded from iPSYCH. ADHD cases in the PGC studies were primarily ascertained from clinics; ADHD was confirmed with structured interviews, and children with comorbid ASD, epilepsy, ID (IQ < 70), and other conditions were excluded. As such, the false positive rate for an ADHD diagnosis is likely higher in the iPSYCH and Swedish register-based datasets than in the PGC studies, while the latter is likely underrepresented for individuals with neurodevelopmental comorbidities. Another major difference is that many of the PGC studies used DSM criteria and thus included children with inattentive and hyperactive-impulsive subtypes of ADHD, whereas the Swedish and iPSYCH studies (and some European PGC studies) used the stricter International Classification of Diseases definition. Despite these differences, we find very high genetic correlation between PGC and iPSYCH, suggesting that overall these diagnostic differences have no perceptible impact on the involvement of common risk variants. PGC-only and iPSYCH-only sex-specific analysis results were similar, with the caveat that the PGC study was smaller and results had larger confidence intervals.

Another possible contribution to sex bias, beyond the scope of the current study, is the role of sex hormones and chromosomes. There is evidence for a specific role of sex hormones (e.g., estrogen) and sex chromosomes (e.g., X chromosome aneuploidy) in early brain development and neurodevelopmental disorders such as ADHD 28, 50, 51, suggesting that future efforts to examine the role of sex chromosomes and their downstream products in ADHD may be worthwhile. Future studies should also examine the degree of shared genetic risks across ADHD and other developmental conditions (e.g., ASD, ID, congenital abnormalities) in a sex-specific manner as well as the role of rare variants (e.g., CNVs, single nucleotide mutations) in ADHD sex bias.

The results of this study demonstrate a clear polygenic contribution from common autosomal genetic variants to ADHD in both sexes, as evidenced by moderate SNP-h^2^ estimates using two methods (similar in both sexes once nonrandom sample ascertainment is taken into account via sample size matching), clear deviation of test statistics on sex-specific GWAS quantile–quantile plots, and significantly higher overall PRSs in cases compared with sex-matched controls. Top hits from sex-specific GWAS analyses corroborate results from a combined analysis of both sexes (7).

The high genetic correlation and lack of difference in polygenic burden between male and female ADHD cases support combining GWAS data from both sexes in ADHD meta-analyses and further suggest that current clinical practices of diagnosing ADHD are capturing a clinical phenotype that is similar at the level of common genetic risk variants in both sexes. Thus, our results indicate that genome-wide autosomal common variants largely do not explain the observed sex bias in ADHD prevalence. On the other hand, the epidemiological results also suggest some degree of clinical heterogeneity, with ADHD showing a stronger phenotypic association with comorbid ASD, congenital malformations, and mild ID in female individuals. We also found evidence for a modest increase in familial risk for ADHD in female individuals based on sibling analysis. Further work simultaneously examining variants across the spectrum of frequencies is needed to comprehensively determine the role of genetic risk in the sex bias in ADHD prevalence.
