# Supplementary material for: A Genetic Investigation of Sex Bias in the Prevalence of Attention-Deficit/Hyperactivity Disorder
Source: Biol Psychiatry. 2018 Jun 15;83(12):1044–53. doi: 10.1016/j.biopsych.2017.11.026 (PMC5992329; doi:10.1016/j.biopsych.2017.11.026)
Supplement: Supplemental Material [file mmc1.pdf]

A Genetic Investigation of Sex Bias in the Prevalence of Attention-Deficit/Hyperactivity Disorder

Supplementary Information

Consortium Members (listed alphabetically)

Psychiatric Genomics Consortium: ADHD Subgroup

Özgür Albayrak, Department of Child and Adolescent Psychiatry, Psychosomatics and Psychotherapy, University Hospital Essen, University of Duisburg-Essen, Essen, Germany; Department of Psychosomatic Medicine and Psychotherapy, Hannover Medical School (MHH), Hannover, Germany; Richard JL Anney, MRC Centre for Neuropsychiatric Genetics and Genomics and Institute of Psychological Medicine and Clinical Neurosciences, Hadyn Ellis Building, Maindy Road, Cathays, Cardiff, UK; Alejandro Arias Vasquez, Radboud University Medical center Nijmegen, Donders Institute for Brain Cognition and Behavior, Departments of Psychiatry, Human Genetics and Cognitive Neuroscience; Maria Jesús Arranz, University Hospital Mutua Terrassa, Barcelona; Philip Asherson, Kings College London; Tobias Banaschewski, Central Institute of Mental Health and Mannheim Medical Faculty, University of Heidelberg; Tobias J. Banaschewski, Department of Child and Adolescent Psychiatry; Central Institute of Mental Health; Medical Faculty Mannheim, University of Heidelberg; Mannheim; Germany; Claiton Bau, Department of Genetics, Instituto de Biociências, Universidade Federal do Rio Grande do Sul, and ADHD outpatient clinic, Hospital de Clínicas de Porto Alegre, Porto Alegre; Joseph Biederman, Massachusetts General Hospital; Harvard Medical School; Preben Bo Mortensen, National Centre for Register-based Research, Aarhus University; Anders Børglum, The Lundbeck Foundation Initiative for Integrative Psychiatric Research, iPSYCH, Denmark. Centre for Integrative Sequencing, iSEQ, Aarhus University, Aarhus, Denmark. Department of Biomedicine – Human Genetics, Aarhus University, Aarhus, Denmark; Jan K. Buitelaar, Radboud University Nijmegen Medical Center; Miguel Casas, Department of Psychiatry, Hospital Universitari Vall d'Hebron. CIBERSAM. Universitat Autònoma de Barcelona and Department of Psychiatry and Legal Medicine, Universitat Autònoma de Barcelona, Catalonia, Spain.; Alice Charach, The Hospital for Sick Children, University of Toronto, Toronto, Ontario, Canada; Bru Cormand, Department de Genètica, Microbiologia i Estadística, Facultat de Biologia, Universitat de Barcelona and Biomedical Network Research Centre on Rare Diseases (CIBERER) and Institut de Biomedicina de la Universitat de Barcelona (IBUB) and Institut de Recerca Sant Joan de Déu (IR-SJD), Barcelona, Catalonia, Spain; Jennifer Crosbie, The Hospital for Sick Children, University of Toronto, Toronto, Ontario, Canada; Jennifer Crosbie, The Hospital for Sick Children, University of Toronto, Toronto, Ontario, Canada; Soeren Dalsgaard, National Centre for Register-based Research, Aarhus University; Mark J. Daly, Analytical and Translational Genetics Unit (ATGU) Massachusetts General Hospital, Boston, MA; Ditte Demontis, The Lundbeck Foundation Initiative for Integrative Psychiatric Research, iPSYCH, Denmark. Centre for Integrative Sequencing, iSEQ, Aarhus University, Aarhus, Denmark. Department of Biomedicine – Human Genetics, Aarhus University, Aarhus, Denmark; Astrid Dempfle, Institute for Medical Informatics and Statistics, University Medical

Center Schleswig-Holstein, Campus Kiel; Alysa E. Doyle, Massachusetts General Hospital; Richard P. Ebstein, National University of Singapore, Singapore 117570; Josephine Elia, duPont Children's hosp, Wilmington, DE; Stephen V. Faraone, Departments of Psychiatry and of Neuroscience and Physiology, SUNY Upstate Medical University, Syracuse, NY, USA; Stephen V. Faraone, SUNY Upstate Medical University; Manuel Föcker, Department of Child and Adolescent Psychiatry, Psychosomatics and Psychotherapy, University Hospital Essen, University of Duisburg-Essen, Essen, Germany; Barbara Franke, Radboud University Medical Centre; Donders Institute for Brain; Cognition and Behaviour; departments of Human Genetics (855) and Psychiatry; Nijmegen; Christine Freitag, Department of Child and Adolescent Psychiatry, Psychosomatics and Psychotherapy, JW Goethe University, 60528 Frankfurt am Main, Germany; Joel Gelernter, Yale; Michael Gill, Department of Psychiatry, Trinity College Dublin, Trinity Centre for Health Sciences, St. James's Hospital, Dublin 8, Ireland; Eugenio Grevet, Department of Psychiatry, Faculdade de Medicina, Universidade Federal do Rio Grande do Sul, and ADHD outpatient clinic, Hospital de Clínicas de Porto Alegre; Jan Haavik, K.G.Jebsen Centre for Neuropsychiatric Disorders, Department of Biomedicine, University of Bergen, Bergen, Norway; Hakon Hakonarson, 1. The Center for Applied Genomics, The Children´s Hospital of Philadelphia, Philadelphia, PA; 2. Department of Pediatrics, The Perelman School of Medicine, University of Pennsylvania, Philadelphia, PA; Ziarih Hawi, School of Psychological Sciences and Monash Institute for Cognitive and Clinical Neurosciences, Monash University; Johannes Hebebrand, Department of Child and Adolescent Psychiatry, Psychosomatics and Psychotherapy, University Hospital Essen, University of Duisburg-Essen, Essen, Germany; Beate Herpertz-Dahlmann, Department of Child and Adolescent Psychiatry, Psychosomatics and Psychotherapy, RWTH Aachen, University Clinics, Aachen, Germany; Amaia Hervas, University Hospital Mutua Terrassa, Barcelona; Anke Hinney, Department of Child and Adolescent Psychiatry, Psychosomatics and Psychotherapy, University Hospital Essen, University of Duisburg-Essen, Essen, Germany; Sarah Hohmann, Central Institute of Mental Health and Mannheim Medical Faculty, University of Heidelberg; Peter Holmans, MRC Centre for Neuropsychiatric Genetics and Genomics, Department of Psychological Medicine and Neurology, School of Medicine, Cardiff University, Cardiff, Wales, CF10 3xQ, UK; Mara Hutz, Department of Genetics, Instituto de Biociências, Universidade Federal do Rio Grande do Sul, Porto Alegre; Abel Ickowitz, The Hospital for Sick Children, University of Toronto, Toronto, Ontario, Canada; Stefan Johansson, K.G.Jebsen Centre for Neuropsychiatric Disorders, Department of Clinical Science, University of Bergen, Bergen, Norway; Lindsey Kent, University of St Andrews; Sarah Kittel-Schneider, Department of Psychiatry, Psychosomatic Medicine and Psychotherapy, University Hospital, Frankfurt Germany; Henry Kranzler,; Jonna Kuntsi, Kings College London; Nanda Lambregts-Rommelse, Karakter Child and Adolescent Psychiatry University Center and department of Psychiatry, Donders Institute for Brain, Cognition and Behavior, Radboud University Nijmegen Medical Centre, Nijmegen, 6500 HB, The Netherlands; Kate Langley, MRC Centre for Neuropsychiatric Genetics & Genomics, School of Medicine, Cardiff University, Cardiff, United Kingdom; Gerd Lehmkuhl, Department of Child and Adolescent Psychiatry, University of Cologne, Cologne, Germany; Klaus-Peter Lesch, "Division of Molecular Psychiatry, ADHD Clinical Research Unit, Department of Psychiatry, Psychosomatics and Psychotherapy, University of Wuerzburg, Germany, and Department of Neuroscience, School for Mental Health and Neuroscience (MHENS), Maastricht University, The Netherlands"; Sandra K. Loo, University of California Los Angeles; Joanna Martin, Department of Medical Epidemiology and Biostatistics, Karolinska Institutet, Stockholm, Sweden; Stanley Center for Psychiatric Research, Broad Institute of MIT and

Harvard, Cambridge, USA; MRC Centre for Neuropsychiatric Genetics and Genomics, Cardiff University, Cardiff, UK; James J. McGough, University of California, Los Angeles, CA; Sarah E. Medland, Queensland Institute of Medical Research; Jobst Meyer, Institute of Psychobiology, Department of Neurobehavioral Genetics, University of Trier, Trier, Germany; Eric Mick, Quantitative Health Sciences University of Massachusetts Medical School, Worcester, MA 01655; Frank Middletion, Department of Neurscience and Physiology, SUNY Upstate Medical University; Ana Miranda, Department of Developmental and Educational Psychology, University of Valencia, Spain; Fernando Mulas, Instituto Valenciano de Neurologia Pediatrica (INVANEP), Valencia, Spain.; Aisling Mulligan, Senior Lecturer in Child and Adolescent Psychiatry, University College Dublin; Benjamin M Neale, Analytical and Translational Genetics Unit (ATGU) Massachusetts General Hospital, Boston, MA; Stan F. Nelson, University of California Los Angeles; T Trang Nguyen, University of Marburg, Marburg, Germany; Michael C. O'Donovan, MRC Centre for Neuropsychiatric Genetics and Genomics and Institute of Psychological Medicine and Clinical Neurosciences, Hadyn Ellis Building, Maindy Road, Cathays, Cardiff, UK; Robert D. Oades, University of Duisburg-Essen; Michael J. Owen, MRC Centre for Neuropsychiatric Genetics and Genomics and Institute of Psychological Medicine and Clinical Neurosciences, Hadyn Ellis Building, Maindy Road, Cathays, Cardiff, UK; Haukur Palmason, Institute of Psychobiology, Department of Neurobehavioral Genetics, University of Trier, Trier, Germany; Josep Antoni Ramos-Quiroga, Department of Psychiatry, Hospital Universitari Vall d'Hebron. CIBERSAM. Universitat Autònoma de Barcelona and Department of Psychiatry and Legal Medicine, Universitat Autònoma de Barcelona, Catalonia, Spain.; Andreas Reif, Department of Psychiatry, Psychosomatic Medicine and Psychotherapy, University Hospital, Frankfurt Germany; Tobias J Renner, Department of Child and Adolescent Psychiatry, Universitätsklinikum Tübingen, Tübingen, Germany; Tobias J Renner, Division of Molecular Psychiatry, ADHD Clinical Research Unit, Department of Psychiatry, Psychosomatics and Psychotherapy, University of Wuerzburg, Germany; Luis Rhode, Department of Psychiatry, Faculdade de Medicina, Universidade Federal do Rio Grande do Sul, and ADHD outpatient clinic, Hospital de Clínicas de Porto Alegre; Marta Ribasés, Department of Psychiatry, Hospital Universitari Vall d'Hebron. CIBERSAM. Universitat Autònoma de Barcelona and Psychiatric Genetics Unit, Vall d'Hebron Research Institute (VHIR), Barcelona, Catalonia, Spain.; Marcella Rietschel, Department of Genetic Epidemiology in Psychiatry; Central Institute of Mental Health; University of Heidelberg; Mannheim; Germany; Stephan Ripke, Analytical and Translational Genetics Unit (ATGU) Massachusetts General Hospital, Boston, MA; Olga Rivero, Division of Molecular Psychiatry, ADHD Clinical Research Unit, Department of Psychiatry, Psychosomatics and Psychotherapy, University of Wuerzburg; Herbert Roeyers, Ghent University, Dunantlaan, Ghent, B-9000 Belgium; Marcel Romanos, University Hospital of Würzburg, Center of Mental Health, Department of Child and Adolescent Psychiatry, Psychosomatics and Psychotherapy; Jasmin Romanos, Department of Child and Adolescent Psychiatry, Psychosomatics and Psychotherapy, University of Wuerzburg, Wuerzburg, 97080 Germany; Nina Roth Mota, ADHD outpatient clinic, Hospital de Clínicas de Porto Alegre, Porto Alegre, RS, Brazil and Department of Human Genetics, Radboud University Medical Center; Aribert Rothenberger, University Medicine Goettingen, Child and Adolescent Psychiatry, Germany; Cristina Sánchez-Mora, Department of Psychiatry, Hospital Universitari Vall d'Hebron. CIBERSAM. Universitat Autònoma de Barcelona and Psychiatric Genetics Unit, Vall d'Hebron Research Institute (VHIR), Barcelona, Catalonia, Spain.; Russell Schachar, Psychiatry, Neurosciences and Mental Health, The Hospital for Sick Children, University of Toronto; Helmut Schäfer, University of Marburg, Marburg, Germany; André

Scherag, Institute for Medical Informatics, Biometry and Epidemiology (IMIBE), University of Duisburg-Essen, Essen, Germany and Clinical Epidemiology, Integrated Research and Treatment Center, Center for Sepsis Control and Care (CSCC), Jena University Hospital, Jena, Germany; Benno G Schimmelmann, University Hospital of Child- and Adolescent Psychiatry, University of Bern, Bern, Switzerland; Joseph Sergeant, Vrije Universiteit, De Boelelaan, Amsterdam, The Netherlands; Judith Sinzig, Department of Child and Adolescent Psychiatry, University of Cologne, Cologne, Germany and Department of Child and Adolescent Psychiatry and Psychotherapy, LVR – Clinic Bonn, Bonn, Germany; Susan L. Smalley, University of California Los Angeles; Edmund J S Sonuga-Barke, University of Southampton and Ghent University, UK; Hans-Christoph Steinhausen, University of Zurich, Switzerland; University of Basel, Switzerland; Child and Adolescent Mental Health Centre, Capital Region Psychiatry, Copenhagen, Denmark; Patrick F Sullivan, Departments of Genetics and Psychiatry, University of North Carolina, Chapel Hill, NC, USA; Department of Medical Epidemiology and Biostatistics, Karolinska Institutet, Stockholm, SE; Anita Thapar, MRC Centre for Neuropsychiatric Genetics and Genomics and Institute of Psychological Medicine and Clinical Neurosciences, Cardiff, United Kingdom; School of Psychology, Cardiff University, Cardiff, United Kingdom; Margaret Thompsom, University of Southampton UK; Alexandre Todorov, Department of Psychiatry, Washington University School of Medicine, St. Louis, Missouri 63110, USA; Irwin Waldman, Emory; Susanne Walitza, Department of Child and Adolescent Psychiatry, University of Zurich, Zurich, CH - 8032, Switzerland and Department of Child and Adolescent Psychiatry, Psychosomatics and Psychotherapy, University of Wuerzburg, Wuerzburg, 97080 Germany; Raymond Walters, Analytical and Translational Genetics Unit (ATGU) Massachusetts General Hospital, Boston, MA; Yufeng Wang, Peking University Institute of Mental Health; Andreas Warnke, Department of Child and Adolescent Psychiatry, Psychosomatics and Psychotherapy, University of Wuerzburg, Wuerzburg, 97080 Germany; Nigel Williams, MRC Centre for Neuropsychiatric Genetics and Genomics, Department of Psychological Medicine and Neurology, School of Medicine, Cardiff University, Cardiff, Wales, CF10 3xQ, UK; Stephanie H Witt, Department of Genetic Epidemiology in Psychiatry; Central Institute of Mental Health; University of Heidelberg; Mannheim; Germany; Li Yang, Peking University Institute of Mental Health; Tetyana Zayats, K.G.Jebsen Centre for Neuropsychiatric Disorders, Department of Biomedicine, University of Bergen, Bergen, Norway; Yanli Zhang-James, Department of Psychiatry, SUNY Upstate Medical University.

iPSYCH-Broad ADHD Workgroup

Esben Agerbo[1,10,11]; Thomas Damm Als[1,2,3]; Marie Bækved-Hansen[1,12]; Rich Belliveau[5]; Anders D. Børglum[1,2,3]; Jonas Bybjerg-Grauholm[1,12]; Felecia Cerrato[5]; Kimberly Chambert[5]; Claire Churchhouse[4,5,13]; Søren Dalsgaard[10]; Mark J. Daly[4,5,13]; Ditte Demontis[1,2,3]; Ashley Dumont[5]; Jacqueline Goldstein[4,5,13]; Jakob Grove[1,2,3,14]; Christine S. Hansen[1,12,15]; Mads Engel Hauberg[1,2,3]; Mads V. Hollegaard [1,12]; David M. Hougaard[1,12]; Daniel P. Howrigan[4,5]; Hailiang Huang[4,5]; Julian Maller[5,16]; Alicia R. Martin[4,5,13]; Joanna Martin[5,6,7]; Manuel Mattheisen[1,2,3,8,9]; Jennifer Moran[5]; Ole Mors[1,21]; Preben Bo Mortensen[1,2,10,11]; Benjamin M. Neale[4,5,13]; Merete Nordentoft[1,19]; Jonatan Pallesen[1,2,3]; Duncan S. Palmer[4,5]; Carsten Bøcker Pedersen[1,10,11]; Marianne Giørtz Pedersen[1,10,11]; Timothy Poterba[4,5,13]; Jesper Buchhave Poulsen[1,12]; Stephan Ripke[4,5,13,17]; Elise B. Robinson[4,18]; F. Kyle Satterstrom[4,5,13]; Christine Stevens[5]; Patrick Turley[4,5]; Raymond K. Walters[4,5]; Thomas Werge[1,15,20]

Affiliations:

[1]The Lundbeck Foundation Initiative for Integrative Psychiatric Research, iPSYCH, Denmark

[2]Centre for Integrative Sequencing, iSEQ, Aarhus University, Aarhus, Denmark

[3]Department of Biomedicine - Human Genetics, Aarhus University, Aarhus, Denmark

[4]Analytic and Translational Genetics Unit, Department of Medicine, Massachusetts General Hospital and Harvard Medical School, Boston, Massachusetts, USA

[5]Stanley Center for Psychiatric Research, Broad Institute of Harvard and MIT, Cambridge, Massachusetts, USA

[6]Department of Medical Epidemiology and Biostatistics, Karolinska Institutet, Stockholm, Sweden

[7]MRC Centre for Neuropsychiatric Genetics & Genomics, School of Medicine, Cardiff University, Cardiff, United Kingdom

[8]Centre for Psychiatry Research, Department of Clinical Neuroscience, Karolinska Institutet, Stockholm, Sweden

[9]Stockholm Health Care Services, Stockholm County Council, Stockholm, Sweden

[10]National Centre for Register-Based Research, Aarhus University, Aarhus, Denmark

[11]Centre for Integrated Register-based Research, Aarhus University, Aarhus, Denmark

[12]Center for Neonatal Screening, Department for Congenital Disorders, Statens Serum Institut, Copenhagen, Denmark

[13]Program in Medical and Population Genetics, Broad Institute of Harvard and MIT, Cambridge, Massachusetts, USA

[14]Bioinformatics Research Centre, Aarhus University, Aarhus, Denmark

[15]Institute of Biological Psychiatry, MHC Sct. Hans, Mental Health Services Copenhagen, Roskilde, Denmark

[16]Genomics plc, Oxford, United Kingdom

[17]Department of Psychiatry, Charite Universitatsmedizin Berlin Campus Benjamin Franklin, Berlin, Germany

[18]Department of Epidemiology, Harvard Chan School of Public Health, Boston, Massachusetts, USA

[19]Mental Health Services in the Capital Region of Denmark, Mental Health Center Copenhagen, University of Copenhagen, Copenhagen, Denmark

[20]Department of Clinical Medicine, University of Copenhagen, Copenhagen, Denmark

[21]Psychosis Research Unit, Aarhus University Hospital, Risskov, Denmark

Supplemental Text

Description of genetic data

Genotype data for ADHD cases and control individuals were available from the Psychiatric Genomics Consortium (PGC) and the Lundbeck Foundation Initiative for Integrative Psychiatric Research (iPSYCH). See GWAS publication for full details (1). The PGC ADHD samples came from a range of studies that were predominantly of European ancestry. They consisted of clinically-ascertained cases of ADHD matched with either controls from the same ancestry group or with pseudo-controls created from the non-transmitted alleles of both parents (trio samples). The individual studies have been previously described in more detail in individual publications (2–16). The iPSYCH sample is based on genotyping of Guthrie cards obtained from the Danish Neonatal Screening Biobank. Blood-spot samples were collected and frozen shortly after birth for individuals born in Denmark and stored in the Danish Newborn Screening Biobank and Statens Serum Institute. The individuals included in the iPSYCH sample were born between May 1, 1981 and December 31, 2005, and had to be alive and resident in Denmark after one year and have a known mother. Cases with ADHD diagnoses (ICD-10 code F90.0) were identified using the Danish Psychiatric Central Research Register. This register includes data on everyone admitted to a psychiatric hospital for assessment or treatment (between 1969 and 2013), as well as everyone who attended psychiatric outpatient services (between 1995 and 2013). Control individuals were randomly selected from the population. The DNA from these samples was extracted, whole-genome amplified in triplicates and genotyped in 23 batches (referred to from here on as waves) using the Illumina PsychChip (a customized HumanCoreExome chip). The first wave consists of the youngest samples (born in 2004) and wave 23 consists of the oldest samples (born in 1981). The study was approved by the Danish Data Protection Agency and the Scientific Ethics Committee in Denmark.

Summary statistics from a GWAS of self-reported ADHD including sex as a covariate were also available from the personal genetics company 23andMe, Inc. Research participants of 23andMe provided informed consent and participated in research online, under a protocol approved by the external AAHRPP-accredited IRB, Ethical & Independent Review Services (E&I Review). The GWAS was based on data from 5,857 self-assessed ADHD cases and 70,393 controls and had a genetic correlation of 0.653 (0.114) with the PGC+iPSYCH samples (1). Results from this GWAS were only used for the polygenic risk score analyses as no raw genotypes or sex-specific summary data were available.

Quality control and data preparation

PGC and iPSYCH samples were processed using the Rapid Imputation Consortium Pipeline (Ricopili), which is a quality control (QC), imputation, and principal components analysis (PCA) pipeline developed and used by the PGC and collaborators. See GWAS publication for full details (1). QC, imputation and PCA were performed separately using the PGC pipeline (Ricopili) for each PGC study and the 23 waves of iPSYCH samples, with the exception that PCA was performed in the entire iPSYCH sample simultaneously. The 1000 Genomes Project, phase 3, data were used as the imputation reference. Cross-study (PGC) and cross-wave (iPSYCH) relatedness analyses were performed in PLINK-v.1.9 on merged, LD-pruned datasets. One of each pair of individuals related at $\hat{\pi} > 0.2$ was excluded (preferentially keeping cases over controls).

See Table S1 for sex-stratified sample sizes for each PGC study and iPSYCH wave. The total sample size after all quality control was N=20,183 cases (25% females) and N=35,191 pseudo-controls/population controls (38% females). Analyses that were restricted to European-only samples consisted of 19,099 cases (26% females) and 34,194 controls (38% females). ADHD GWAS summary statistics were also available from research participants of the personal genetics company 23andMe, Inc. (N=5,857 self-reported ADHD cases, 70,393 controls).

Sex-specific GWAS analyses

Sex-specific case-control genome-wide logistic regression analyses of imputed autosomal dosage data were performed in each PGC study and iPSYCH wave separately, using the "--dosage" option in PLINK-version-1.9, co-varying for principal components (PCs) and/or site indicator variables, as appropriate. iPSYCH samples included the first 4 PCs and any PCs significantly associated with case status, obtained from the joint PCA in the entire iPSYCH sample. For PGC studies with <1000 samples, the top 5 PCs were used and for studies with ≥1000 samples, the first 10 PCs were used as covariates. For the IMAGE-1 study, indicator variables coding for site ID were included as covariates instead of PCs, as this study used a trio design but consisted of samples contributed by several different data collection sites. Trio studies were split by case sex, keeping each pseudo-control together with its corresponding case.

Results were filtered for each study/wave and SNPs meeting the following criteria were retained for the sex-specific analyses: imputation quality (INFO score) > 0.8, call rate in best guess genotype data > 0.925, minor allele frequency (MAF) > 0.01, and expected MAF in cases (2 x MAF in controls x no. of cases) > 1. Sex-specific GWAS meta-analyses of filtered results were performed in METAL using the standard error analysis scheme (STDERR).

Meta-analysis results were additionally filtered to retain only SNPs that were available for analysis in at least half of the total sample size and present in both the male-only and female-only analyses. This yielded results for N=7,531,543 common variants in the meta-analyses (hereafter: PGC+iPSYCH).

Estimating SNP-heritability and genetic correlation

Bivariate LD score regression (using LDSC (17,18)) analyses were run on the sex-specific meta-analyzed summary statistics. The primary analyses (with the most power) are those for the full PGC+iPSYCH sample but we also examined estimates in the PGC and iPSYCH samples separately using LDSC and a second method, GREML (using GCTA (19,20)), to examine the stability of the findings. Sex-specific heritability was also estimated using univariate models. Analyses were restricted to European-only samples.

LD scores from a European reference panel provided with the LDSC software were used for analysis. LDSC analyses were based on the following numbers of SNPs, after restriction to HapMap SNPs: PGC-only: 1,108,369 SNPs; iPSYCH-only: 1,021,086 SNPs; PGC+iPSYCH 1,023,856 SNPs. The intercept was not constrained in LDSC, to provide unbiased estimation. For sensitivity, genetic correlation analyses were also run in LDSC to assess cross-dataset (PGC vs. iPSYCH) within- and across-sex genetic correlations.

Because of strict restrictions on access to individual genotypes, bivariate GREML analyses were only performed separately in the PGC and iPSYCH samples. For each of these datasets, best guess genotype data were generated using Ricopili and strictly filtered (MAF>0.05, in addition to previous frequency, imputation quality and other filters). Genotypes were merged together across studies using PLINK. Asymmetric/ambiguous (AT, TA, CG, GC), multi-allelic and duplicate position SNPs were excluded. For each dataset, a genomic-relationship matrix was calculated using GCTA, restricted to HapMap-3 SNPs. Analyses were based on the following numbers of SNPs: PGC-only: 191,466 SNPs; iPSYCH-only: 435,086 SNPs. One of each pair of individuals related at the level of second cousins (pi-hat>0.05) was excluded, preferentially keeping cases; this excluded: N=16 cases and N=91 controls in the PGC dataset and N=1,439 cases and N=3,170 controls in the iPSYCH dataset. PCA (after LD-pruning and removing SNPs located in long-range LD regions) was performed on the merged, unrelated samples using PLINK, to derive population covariates. The first 10 PCs as well as binary study/wave indicators were used as covariates for subsequent analyses. Bivariate GREML was used to estimate the genetic correlation across-sex. Univariate GREML analyses in GCTA were used to estimate SNP-h^2 in males and females with ADHD relative to sex-matched controls.

The expected range of the genetic correlation (r_g) estimates should be from -1 to 1. However, the estimator was left unconstrained for these analyses in GREML and LDSC to allow for an unbiased assessment of the standard errors of the estimates; as such, some of the estimates exceed this range. Specific tests were used to determine whether the SNP-h^2 (on the liability scale) estimates differed significantly for males and females using the formula: $(\text{SNP-}h^2_F - \text{SNP-}h^2_M)^2/(SE_F^2 + SE_M^2)$ with a Chi^2 test with 1 degree of freedom. One-tailed tests were also used to determine whether the estimates of genetic correlation differed significantly from one ($z=(1-r_g)/SE$) or from zero ($z=r_g)/SE$), compared to a normal distribution.

Based on an estimated population prevalence rate of approximately 5% (21) for ADHD and an observed male:female ratio of approximately 3:1 in the cases, the following prevalence rates were assumed for converting the estimates of SNP-heritability to the liability scale: 2.5% in females and 7.5% in males. Analyses were also re-run assuming different relative population prevalence for males and females, depending on the assumed ratio of the relative prevalence (ranging from equal prevalence assumed to a 7:1 male bias). This was done to examine the sensitivity of this assumption on the estimation of liability scale SNP-h^2. These analyses were also repeated while randomly down-sampling the number of male cases and controls to match the available sample size for females within each study/wave.

Secondary GWAS analyses

A number of secondary GWAS analyses were run to further examine the impact of sex on genome-wide association analyses of ADHD. First, heterogeneity statistics from a meta-analysis of the male-only and female-only summary statistics were examined for all SNPs. Second, combined GWAS analyses including a sex-by-genotype interaction term were carried out. Third, the genetic correlation was estimated using LDSC for GWAS analyses of the combined sample including and excluding sex as a covariate. Finally, GWAS analyses of case sex (male cases coded as 0 and female cases coded as 1) were carried out.

Polygenic risk score analyses

A leave-one-study/wave-out approach was used to maximize power and maintain fully independent target and discovery samples for polygenic risk score (PRS) analyses, using the standard approach (22,23). First, GWAS analyses of imputed dosage data were run for all samples in each PGC study and iPSYCH wave separately, as described previously, co-varying for PCs as well as sex. Meta-analyses using METAL (with the STDERR scheme) were run excluding one set of summary results at a time, for each combination of studies. To maximize power for the discovery samples, GWAS results from 23andMe and non-European samples were also included in the ADHD discovery meta-analyses. For each set of discovery results, LD-clumping was run to obtain a relatively independent set of SNPs, while retaining

the most significant SNP in each LD block. The following parameters were applied in PLINK: --clump-kb 500 --clump-r2 0.3 --clump-p1 0.5 --clump-p2 0.5. Asymmetric/ambiguous (AT, TA, CG, GC) SNPs, indels, multi-allelic and duplicate position SNPs were excluded. The SNP selection p-value threshold used was p<0.1. The number of clumped SNPs for each study/wave varied from 20,596-43,427 (see Table S9).

PRS were calculated for each individual in the independent target sample (restricted to European samples) by scoring the number of risk alleles (weighted by the SNP log of the odds ratio) across the set of clumped, meta-analyzed SNPs in PLINK.v.1.9 (using the command --score no-mean-imputation). Scores were derived in best guess genotype data after filtering out SNPs with MAF<0.05 and INFO<0.8. The PRS were standardized using z-score transformations; odds ratios can be interpreted as the increase in risk of the outcome, per standard deviation in PRS. Logistic regression analyses including PCs tested for association of PRS with sex in the cases (males were coded as 0 and females were coded as 1) and case status, stratified by sex. Overall meta-analyses of the leave-one-out analyses were performed. The analyses were re-run using European-only samples and then also by excluding sex as a covariate in the discovery GWAS analyses, as sensitivity tests. Analyses were also performed on sex in controls as well as sex of parents in studies using a parent-offspring trio design. All regression and meta-analyses were run in R-3.2.2.

Epidemiological analyses

Analyses of Swedish registry data were based on all individuals born in Sweden between 1987 and 2006, as identified using the Medical Birth Register. Data linkage of several nation-wide Swedish registers was performed using the unique personal identification number (24). Information from the Total Population Register (25), Cause of Death Register and the Multi-Generation Register (26) was used to identify those individuals of known maternity and paternity who lived in Sweden at least until age 12 years (or until the time of this study, if they were younger than 12 years old). Information on ADHD diagnoses was obtained from the National Patient Register (27) for ICD-9 (1987-1996) and ICD-10 (1997-2013) and from the Prescribed Drug Register (28) (June 2005-2014). ADHD cases were defined as those individuals who had at least 2 recorded diagnoses of ADHD or 2 recorded prescriptions of ADHD medication (Methylphenidate, Amphetamine, Dexamphetamine, Atomoxetine or Lisdexamfetamine) after the age of 3 years. Analyses were based on N=77,905 ADHD cases and N=1,874,637 control individuals. The data linkage of the Swedish registry data was approved by the regional ethics review board in Stockholm, Sweden. The requirement for informed consent was waived, because the study was register-based, and individuals were not personally identifiable at any time.

All epidemiological analyses were performed in R (with the ‘drgee’ package). Children were clustered together if they shared the same biological mother, in order to obtain standard errors that accounted for non-independent observations. Birth year was included as a covariate in all analyses.

We assessed whether females affected with ADHD are at a higher risk than males for comorbid severe developmental disorders and rare genetic syndromes. International Classification of Diseases (ICD) codes for the following categories of disorders were examined: intellectual disability (ID), autism spectrum disorder (ASD), developmental coordination disorder (DCD), epilepsy, congenital malformations (CM) and chromosomal abnormalities (CA); see Table S10 for specific ICD codes. Diagnoses of ASD and DCD were only considered after age 1 year and ID diagnoses after age 2 years. No age restrictions were made for epilepsy, CM or CA. For each comorbid condition, individuals were considered as affected (coded as 1) if they had at least 2 recorded diagnoses in that category and unaffected (coded as 0) if they did not meet these criteria. Generalized estimating equations were used to test for the effect of an ADHD-by-sex interaction term on each outcome. First, the effect of presence of ADHD on each outcome within individuals was estimated separately for males and females (analytic model: gee(outcome ~ ADHD + birth_year)). Next, we tested for an ADHD-by-sex interaction term on each outcome, using the following analytic model: gee(outcome ~ ADHD + sex + ADHD*sex + birth_year). For individuals with available information on severity of ID, secondary analyses were run for 3 severity categories: mild, moderate and severe/profound.

To test the female protective effect hypothesis, we estimated whether risk of ADHD in siblings of females with ADHD was higher than for siblings of affected males, stratified by the sex of the comparison sibling. Analyses were restricted to pairs of full siblings, based on sharing both biological parents). Twins (i.e. children born with 2-weeks of each other) were excluded as zygosity could not be confirmed. Analyses were restricted to sibling pairs with at least 1 child who had a diagnosis of ADHD, as defined above (N=71,691 observations (of which, N=23,452 came from female probands), consisting of N=21,784 unique index individuals, of which N=7,186 came from unique female probands). The effect of the proband being female on the comparison sibling’s risk for ADHD was estimated using the following model, stratified by the sex of the comparison sibling: gee(ADHD_sib2 ~ sex_sib1 + birth_year_sib2).

Test of equality of variances of ADHD PRS by case sex

Levene’s Test was used to determine whether the variance in ADHD PRS differed between male and female cases. For each study/wave, PRS were residualised for covariates. Levene’s Test was applied (using the ‘car’ package in R), centering the data by mean in each

group. To perform meta-analysis of the Levene's Test, we transformed each wave/study p-value into a Z score, assigning the sign of the Z with increased variance in males being negative. We then summed the per wave Z-scores and divided by the square root of the number of studies to obtain a meta-analytic Z, which we then tested. The combined Z-score for these tests was -1.02 with a p-value of 0.31.

Table S1: Sample size and description of individual ADHD studies and iPSYCH waves

Sample		Cases				Controls				Age	Design	Ancestry
		Females	Males	Total	% F	Females	Males	Total	% F			
PGC	Barcelona, Spain	173	399	572	0.30	100	325	425	0.24	A	Case-control	European
	Beijing, China	159	853	1012	0.16	350	575	925	0.38	C	Case-control	Han Chinese
	Bergen, Norway	158	137	295	0.54	123	79	202	0.61	A	Case-control	European
	Cardiff, UK	93	628	721	0.13	2511	2570	5081	0.49	C	Case-control	European
	CHOP	64	198	262	0.24	64	198	262	0.24	C	Trios	European
	Germany	94	393	487	0.19	634	656	1290	0.49	C	Case-control	European
	IMAGE-I	85	615	700	0.12	85	615	700	0.12	C	Trios	European
	IMAGE-II	116	508	624	0.19	877	878	1755	0.5	C	Case-control	European
	PUWMa	227	408	635	0.36	227	408	635	0.36	C	Trios	Diverse (USA)
	PUWMa (strict)	202	361	563	0.36	202	361	563	0.36	C	Trios	European
	Toronto, Canada	27	82	109	0.25	27	82	109	0.25	C	Trios	European
	Yale-Penn	55	127	182	0.30	555	760	1315	0.42	A	Case-control	European
iPSYCH (Denmark)	Wave 1	307	1066	1373	0.22	488	528	1016	0.48	C & A	Case-control	European
	Wave 2	233	938	1171	0.20	1311	1322	2633	0.50	C & A	Case-control	European
	Wave 3	186	761	947	0.20	513	512	1025	0.50	C & A	Case-control	European
	Wave 4	128	490	618	0.21	500	526	1026	0.49	C & A	Case-control	European
	Wave 5	196	583	779	0.25	503	479	982	0.51	C & A	Case-control	European
	Wave 6	188	588	776	0.24	456	515	971	0.47	C & A	Case-control	European
	Wave 7	151	514	665	0.23	393	468	861	0.46	C & A	Case-control	European
	Wave 8	216	601	817	0.26	544	539	1083	0.50	C & A	Case-control	European
	Wave 9	215	598	813	0.26	495	538	1033	0.48	C & A	Case-control	European
	Wave 10	168	352	520	0.32	359	388	747	0.48	C & A	Case-control	European
	Wave 11	185	407	592	0.31	382	390	772	0.49	C & A	Case-control	European
	Wave 12	143	282	425	0.34	379	421	800	0.47	C & A	Case-control	European
	Wave 13	140	319	459	0.31	391	419	810	0.48	C & A	Case-control	European
	Wave 14	158	312	470	0.34	383	395	778	0.49	C & A	Case-control	European
	Wave 15	164	417	581	0.28	438	418	856	0.51	C & A	Case-control	European
	Wave 16	169	376	545	0.31	426	401	827	0.52	C & A	Case-control	European
	Wave 17	166	332	498	0.33	412	388	800	0.52	C & A	Case-control	European
	Wave 18	135	281	416	0.32	446	467	913	0.49	C & A	Case-control	European
	Wave 19	145	263	408	0.36	464	451	915	0.51	C & A	Case-control	European
	Wave 20	90	223	313	0.29	443	406	849	0.52	C & A	Case-control	European
	Wave 21	135	308	443	0.30	419	445	864	0.48	C & A	Case-control	European
	Wave 22	100	213	313	0.32	455	476	931	0.49	C & A	Case-control	European
	Wave 23	160	482	642	0.25	468	532	1000	0.47	C & A	Case-control	European
23andMe		N/A	N/A	5857	N/A	N/A	N/A	70393	N/A	A	Case-control	Diverse (USA)

**TOTAL PGC+iPSYCH (European-only)**	**4945**	**14154**	**19099**	**0.26**	**16246**	**17948**	**34194**	**0.38**			
**TOTAL PGC+iPSYCH (All ancestries)**	**5129**	**15054**	**20183**	**0.25**	**16621**	**18570**	**35191**	**0.38**			
**GRAND TOTAL (including 23andMe)**			**26040**				**105584**				

A: adults; C: children

Table S2: The number of clumped SNPs in each study/wave

Study/wave	Number of clumped SNPs
**PGC**	
Barcelona, Spain	43427
Bergen, Norway	41417
Cardiff, UK	39110
CHOP	38574
Germany	38406
IMAGE-I	34930
IMAGE-II	20596
PUWMa	37751
PUWMa (strict)	34469
Toronto, Canada	30549
Yale-Penn	43427
**iPSYCH**	
Wave 1	27638
Wave 2	27270
Wave 3	27494
Wave 4	27635
Wave 5	27051
Wave 6	27165
Wave 7	27347
Wave 8	27444
Wave 9	27950
Wave 10	27980
Wave 11	27975
Wave 12	27605
Wave 13	28280
Wave 14	28258
Wave 15	28546
Wave 16	28457
Wave 17	28397
Wave 18	28703
Wave 19	28551
Wave 20	28470
Wave 21	28515
Wave 22	28443
Wave 23	27554

Table S3: ICD codes for neurodevelopmental disorders examined in relation to ADHD and sex

Category	Diagnosis	ICD-10 codes	ICD-9 codes
**ADHD**	Attention deficit hyperactivity disorder	F90	314
**ASD**	Autism spectrum disorder	F84	299
**DCD**	Developmental coordination disorder	F82	315.4
**Intellectual disability**	Mental retardation	F70-F79	317-319
**Epilepsy**	Epilepsy	G40	345
	Status Epilepticus	G41	345Q
	Epilepsy-aphasia	F80.3	
**Congenital malformations**	Craniofacial dysmorphia (including neck)	Q10-Q18	743-744
	Other congenital abnormalities	Q00-Q07, Q20-Q89	740-742, 745-757, 759
**Chromosomal abnormalities**	Down's Syndrome	q90	758
	Edward's syndrome and Patau syndrome	q91	758.1,758.2
	Other autosomal trisomies	q92	758.5
	Autosomal monosomies	q93	758.3,758.5
	Translocations disorders	q95	758.4,758.5
	Turner's syndrome	q96	758.6
	Other female sex chromosome defects	q97	758.81
	Other male sex chromosome defects	q98	758.7,758.81
	Other chromosome defects	q99	758.81-758.89

Abbreviations: ADHD: attention deficit hyperactivity disorder; ASD: autism spectrum disorder; DCD: developmental coordination disorder.

Table S4: SNP-heritability estimates for females, males and the combined sample and genetic correlation estimates for females and males, using GREML and LDSC

Method	Sample	Sex	Liability scale		Observed scale		Intercept	Intercept SE	M vs F Rg	M vs F Rg (SE)	p=0	p=1
			SNP-h^2	SNP-h^2 SE	SNP-h^2	SNP-h^2 SE						
**LDSC**	**PGC+ iPSYCH**	Females	0.123	0.025	0.125	0.025	1.062	0.008	1.220	0.132	1.1E-20	0.95
		Males	0.247	0.021	0.253	0.021	1.012	0.008				
		All	0.218	0.015	0.237	0.016	1.035	0.010				
	**iPSYCH**	Females	0.157	0.034	0.173	0.038	1.054	0.009	1.202	0.156	6.8E-15	0.90
		Males	0.294	0.024	0.306	0.025	1.007	0.008				
		All	0.259	0.018	0.290	0.020	1.027	0.009				
	**PGC**	Females	0.276	0.084	0.224	0.068	0.971	0.007	0.626	0.265	9.1E-03	0.079
		Males	0.159	0.052	0.151	0.049	1.004	0.006				
		All	0.114	0.032	0.109	0.031	1.013	0.006				
**GREML**	**iPSYCH**	Females	0.159	0.017	0.189	0.020			0.952	0.067	4.3E-46	0.24
		Males	0.210	0.014	0.218	0.014						
		All	0.187	0.008	0.215	0.010						
	**PGC**	Females	0.087	0.035	0.071	0.029			1.065	0.266	3.2E-05	0.60
		Males	0.112	0.020	0.106	0.019						
		All	0.104	0.013	0.098	0.013						

F: females; M: males. Population prevalence rates assumed for ADHD were: 2.5% in females, 7.5% in males and 5% in the combined sample. Intercepts and associated standard errors are only available for LDSC. p=0 refers to a test of whether the estimate differs significantly from zero; p=1 refers to a test of whether the estimate differs significantly from 1.

Table S5: Cross-dataset and cross-sex LDSC genetic correlation estimates

Analysis	Phenotype 1	Phenotype 2	rg	se	p=0	p=1
**Cross-dataset**	PGC F+M	iPSYCH F+M	1.133	0.220	1.3E-07	0.73
**Cross-dataset, within-sex**	PGC F	iPSYCH F	0.316	0.173	0.034	3.8E-05
	PGC M	iPSYCH M	1.068	0.228	1.4E-06	0.62
**Cross-dataset, cross-sex**	PGC M	iPSYCH F	1.029	0.281	1.3E-04	0.54
	PGC F	iPSYCH M	0.731	0.158	1.7E-06	0.044

F: females; M: males; p=0 refers to a test of whether the estimate differs significantly from zero; p=1 refers to a test of whether the estimate differs significantly from 1.

Table S6: SNP-heritability estimates for males, after down-sampling cases and controls to match available sample size in females

Method	Sample	Sex	Liability scale		Observed scale		Intercept	Intercept SE
			SNP-h^2	SNP-h^2 SE	SNP-h^2	SNP-h^2 SE		
**LDSC**	**PGC+ iPSYCH**	Males	0.131	0.039	0.097	0.029	1.067	0.009
	**iPSYCH**	Males	0.216	0.047	0.173	0.037	1.049	0.008
	**PGC**	Males	0.199	0.123	0.117	0.072	0.983	0.006
**GREML**	**iPSYCH**	Males	0.215	0.024	0.185	0.020		
	**PGC**	Males	0.076	0.047	0.045	0.028		

Population prevalence rate assumed for ADHD in males was 7.5%.

Table S7: SNP-heritability estimates for females and males, varying the assumed population prevalence based on different male:female ratios

M:F ratio	Proportion		Prevalence		SNP-h^2 (SE)			p	p*
	M	F	M	F	M	F	M (matched N)		
1	0.50	0.50	0.050	0.050	0.218 (0.018)	0.150 (0.030)	0.116 (0.034)	0.055	0.45
2	0.67	0.33	0.067	0.033	0.238 (0.020)	0.133 (0.027)	0.126 (0.038)	1.6E-03	0.89
3	0.75	0.25	0.075	0.025	0.247 (0.021)	0.123 (0.025)	0.131 (0.039)	1.3E-04	0.86
4	0.80	0.20	0.080	0.020	0.252 (0.021)	0.116 (0.024)	0.134 (0.040)	1.5E-05	0.70
5	0.83	0.17	0.083	0.017	0.255 (0.021)	0.111 (0.023)	0.135 (0.040)	3.1E-06	0.60
6	0.86	0.14	0.086	0.014	0.258 (0.021)	0.106 (0.022)	0.137 (0.041)	5.5E-07	0.50
7	0.88	0.13	0.088	0.013	0.260 (0.022)	0.104 (0.021)	0.138 (0.041)	2.4E-07	0.46

F: females; M: males. Estimates are based on the full PGC+iPSYCH dataset and were obtained using LDSC. P-values displayed are for a test of whether the SNP heritability differs significantly in males and females; *difference between female SNP-h^2 estimates and estimates in males matched for sample size.

Table S8: Top 10 LD-independent SNPs for male-only and female-only GWAS meta-analyses

**Female-only GWAS Top SNPs**										**Top SNPs from female-only GWAS in male-only GWAS results**			
**SNP**	**CHR**	**BP**	**A1**	**A2**	**Beta**	**SE**	**P**	**N**	**Nearest gene (distance)**	**Beta**	**SE**	**P**	**N**
rs7181782	15	80686993	C	G	-0.170	0.032	9.3E-08	22069	ARNT2(-9.698kb)	-0.007	0.022	0.73	31224
rs4984687	16	754314	T	C	-0.168	0.032	1.9E-07	19486	FBXL16 (0kb)	-0.021	0.022	0.33	27862
rs28600876	11	69705480	C	G	0.134	0.027	6.5E-07	20466	FGF3(+71.29kb)	-0.010	0.019	0.59	28951
rs114247285	5	75339982	A	G	0.339	0.069	7.7E-07	14236	SV2C(-39.26kb)	-0.023	0.046	0.62	21421
rs9941217	16	18050926	C	G	0.128	0.026	9.3E-07	22069	NA	0.028	0.018	0.11	31224
rs142295881	6	53899088	A	G	-0.465	0.095	1.0E-06	20237	MLIP(0)	-0.062	0.066	0.35	29242
rs79891548	2	166337818	T	C	-0.216	0.044	1.1E-06	21459	CSRNP3(0)	-0.026	0.031	0.41	30337
rs150844750	2	109359283	T	C	-0.250	0.052	1.5E-06	22069	RANBP2(0)	-0.058	0.036	0.11	31224
rs71450738	12	24198831	T	TCCATAG	-0.118	0.025	1.7E-06	22069	SOX5(0)	-0.023	0.017	0.17	31224
rs770082	12	89776485	A	G	0.120	0.025	1.8E-06	22069	DUSP6(+29.85kb)	0.062	0.017	2.9E-04	31224
**Male-only GWAS Top SNPs**										**Top SNPs from male-only GWAS in female-only GWAS results**			
**SNP**	**CHR**	**BP**	**A1**	**A2**	**Beta**	**SE**	**P**	**N**	**Nearest gene (distance)**	**Beta**	**SE**	**P**	**N**
rs3047819	5	88175199	T	TTA	-0.097	0.017	1.5E-08	30337	MEF2C(0)	-0.013	0.025	0.61	21459
rs8039398	15	47730870	T	C	-0.094	0.017	2.6E-08	31224	SEMA6D(0)	-0.049	0.025	0.048	21960
rs200508662	5	120391182	T	C	0.131	0.024	3.1E-08	31224	NA	0.010	0.035	0.78	22069
rs145108385	5	43054747	A	G	0.098	0.019	1.8E-07	30337	LOC648987(0)	0.035	0.027	0.19	21459
rs112984125	1	44173423	A	G	-0.097	0.019	1.9E-07	31224	ST3GAL3(0)	-0.102	0.027	1.8E-04	22069
rs142458453	12	31530325	T	TTAAATAAA	0.104	0.021	4.6E-07	31224	DENND5B(-4.831kb)	-0.054	0.030	0.074	22069
rs56135409	3	20725016	A	C	-0.087	0.017	5.5E-07	31224	NA	-0.044	0.025	0.083	22069
rs11317767	17	8674141	G	GT	0.087	0.017	6.1E-07	31224	SPDYE4(+12.26kb)	-0.004	0.025	0.87	22069
rs9746958	16	60618639	A	G	0.311	0.063	6.7E-07	30337	NA	0.070	0.100	0.48	19603
rs74129883	10	48328126	A	C	-0.218	0.044	6.8E-07	21403	ZNF488(-26.96kb)	-0.033	0.073	0.65	12378

Results are LD-clumped using PLINK (--clump-kb 3000 --clump-r2 0.1 --clump-p1 0.0001 --clump-p2 0.01) including the 1KG as the reference and annotated using a gene region list in PLINK. Results are sorted by p-value in the discovery sample.

Table S9: Frequency and proportion of severe neurodevelopmental outcomes stratified by ADHD case status and sex in the Swedish population samples (Total N=1,952,542)

Outcome	Full sample	ADHD cases		Controls	
		Females (N=25,832)	Males (N=52,073)	Females (N=923,895)	Males (N=950,742)
**ASD**	22134 (1.13)	3111 (12.04)	7718 (14.82)	3768 (0.41)	7537 (0.79)
**DCD**	2841 (0.15)	280 (1.08)	967 (1.86)	622 (0.07)	972 (0.10)
**ID**	13318 (0.68)	1264 (4.89)	2760 (5.30)	4042 (0.44)	5252 (0.55)
**Epilepsy**	16921 (0.87)	737 (2.85)	1337 (2.57)	7286 (0.79)	7561 (0.80)
**CA**	100844 (5.16)	1670 (6.46)	4270 (8.20)	40148 (4.35)	54756 (5.76)
**CM**	4319 (0.22)	134 (0.52)	312 (0.60)	1956 (0.21)	1917 (0.20)

The table displays exact N and proportion of each group as N (%). Abbreviations: ADHD: attention deficit hyperactivity disorder; ASD: autism spectrum disorder; CA: chromosomal abnormalities; CM: congenital malformations; DCD: developmental coordination disorder; ID: intellectual disability.

Table S10: Results of secondary logistic regression analyses of ADHD on intellectual disability, depending on severity, in the Swedish population sample

Outcome	Sex	% of ADHD cases with outcome	Sex-specific association				ADHD*sex interaction			
			OR	LCI	UCI	p	OR	LCI	UCI	p
**Mild ID**	**Males**	3.84	14.89	14.04	15.79	<2.2E-308	1.13	1.02	1.24	0.014
	**Females**	3.84	16.79	15.56	18.11	<2.2E-308				
**Moderate ID**	**Males**	0.55	6.94	6.07	7.94	3.1E-177	0.97	0.76	1.23	0.809
	**Females**	0.45	6.74	5.52	8.23	1.3E-78				
**Severe/ profound ID**	**Males**	0.15	2.79	2.21	3.53	7.1E-18	0.66	0.41	1.06	0.0873
	**Females**	0.09	1.84	1.22	2.78	3.6E-03				

Abbreviations: ADHD: attention deficit hyperactivity disorder; ID: intellectual disability.

Table S11: Results of regressions of ADHD PRS and sex of parents in studies with parent-offspring trio designs

Sample	OR	LCI	UCI	P	N
CHOP	1.15	0.96	1.37	0.12	524
Canada	1.16	0.88	1.54	0.29	218
IMAGE-1	1.01	0.90	1.12	0.90	1400
PuWMa	0.99	0.88	1.11	0.81	1126

Mothers are coded as 1 and fathers are coded as 0 in logistic regression analyses.

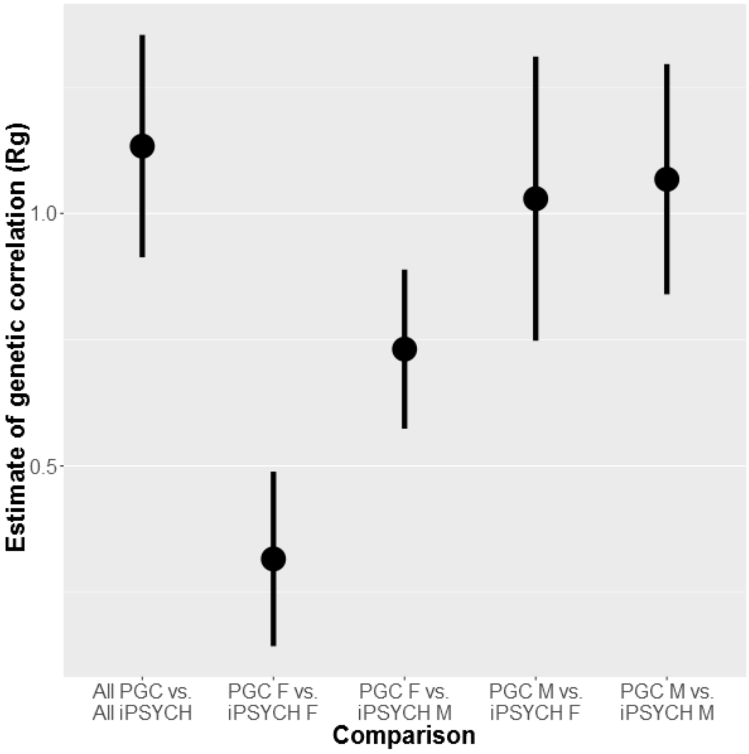

Figure S1

Genetic correlation estimates for ADHD in the iPSYCH and PGC datasets, within and across sex. F: female; M: male. Confidence intervals display standard errors.

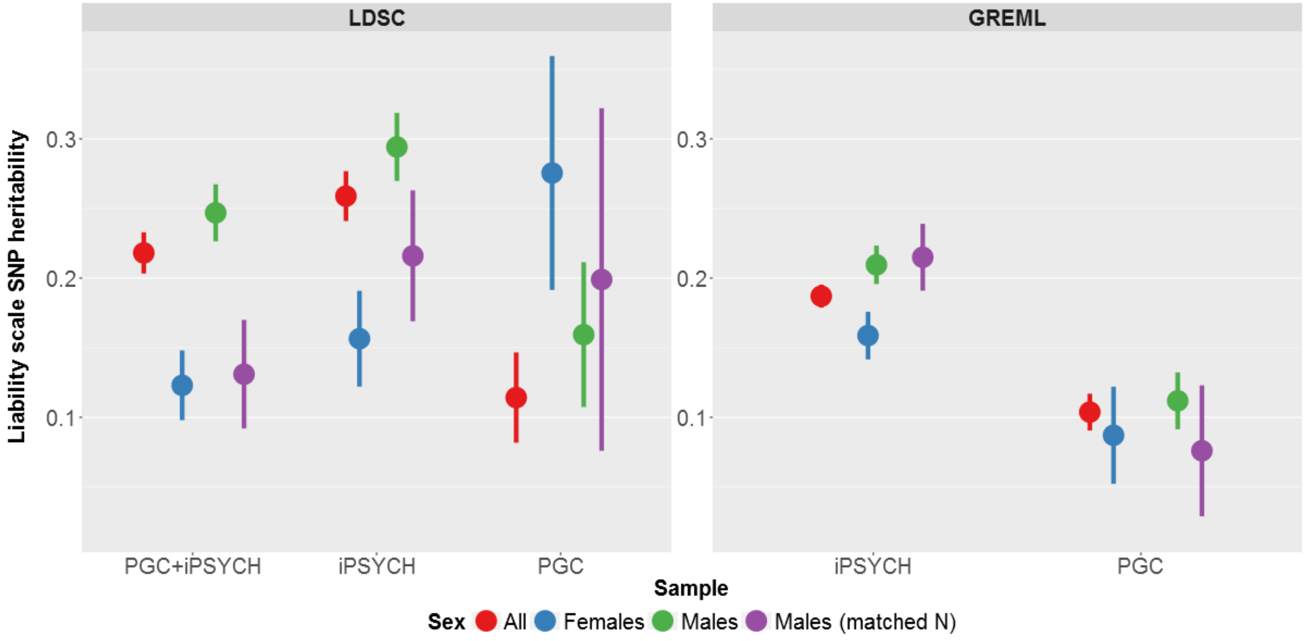

Figure S2

SNP-heritability estimates on the liability scale obtained from univariate GREML models for the iPSYCH and PGC datasets and LDSC analyses in the iPSYCH, PGC and combined PGC+iPSYCH datasets. Estimates are shown for the whole sample and for females and males separately, as well as for a restricted sample of males matched for sample size (N) to females. Population prevalence rates assumed for ADHD were: 2.5% in females, 7.5% in males and 5% in the combined sample. Because of strict restrictions on raw individual genotype access and transfer, GREML analyses could only be performed separately in the PGC and iPSYCH samples. Confidence intervals display standard errors.

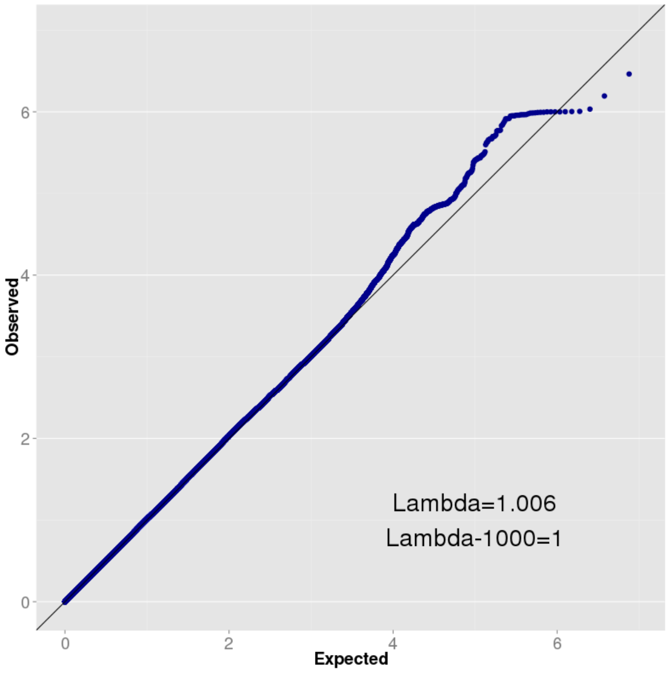

Figure S3

QQ plot of heterogeneity statistics from a meta-analysis of the male-only and female-only summary statistics.

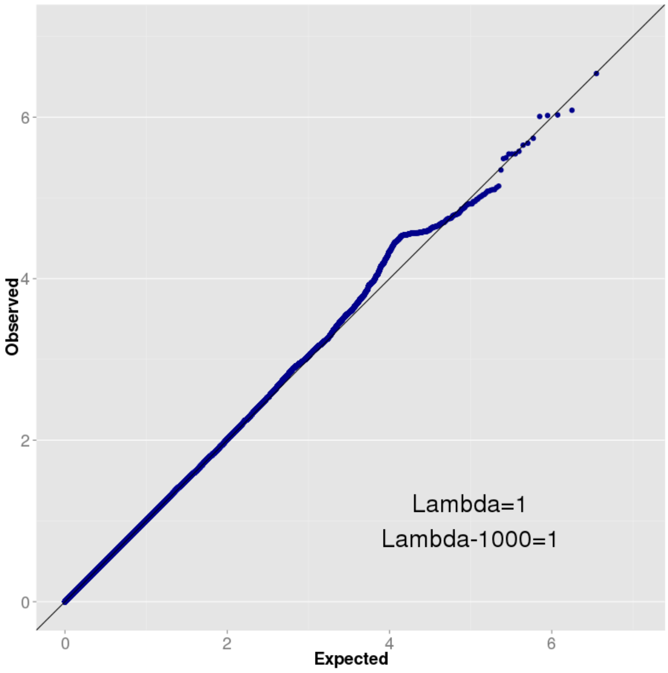

Figure S4

QQ plot of sex-by-genotype interaction terms in a combined male and female GWAS analysis.

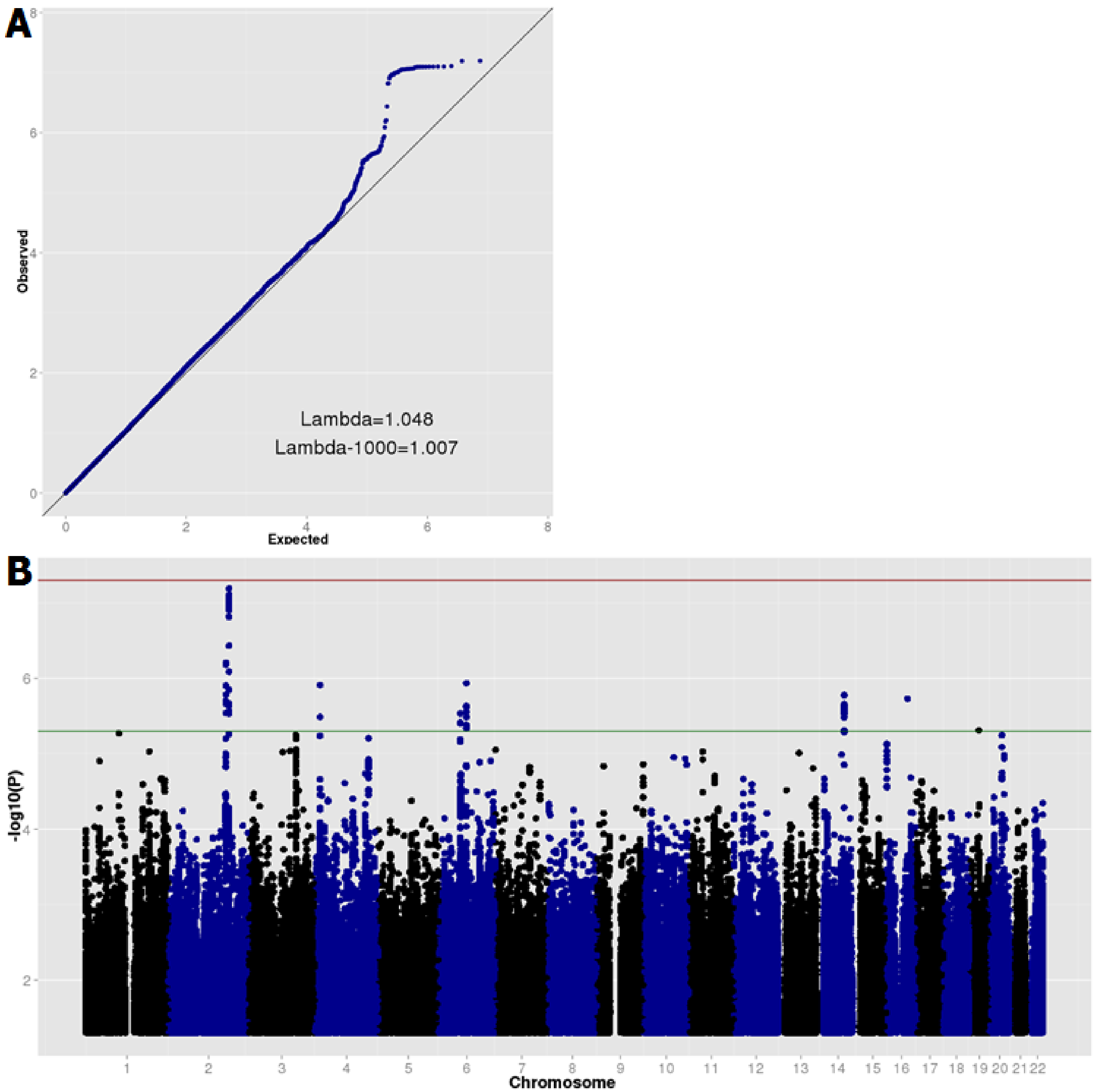

Figure S5

QQ plot (a) and Manhattan plot (b) for GWAS analyses of case sex (male cases coded as 0 and female cases coded as 1). In figure(b) the horizontal red line indicates genome-wide significance (p<5E-8) and the horizontal green line indicates suggestive sub-threshold signals (p<5E-6).

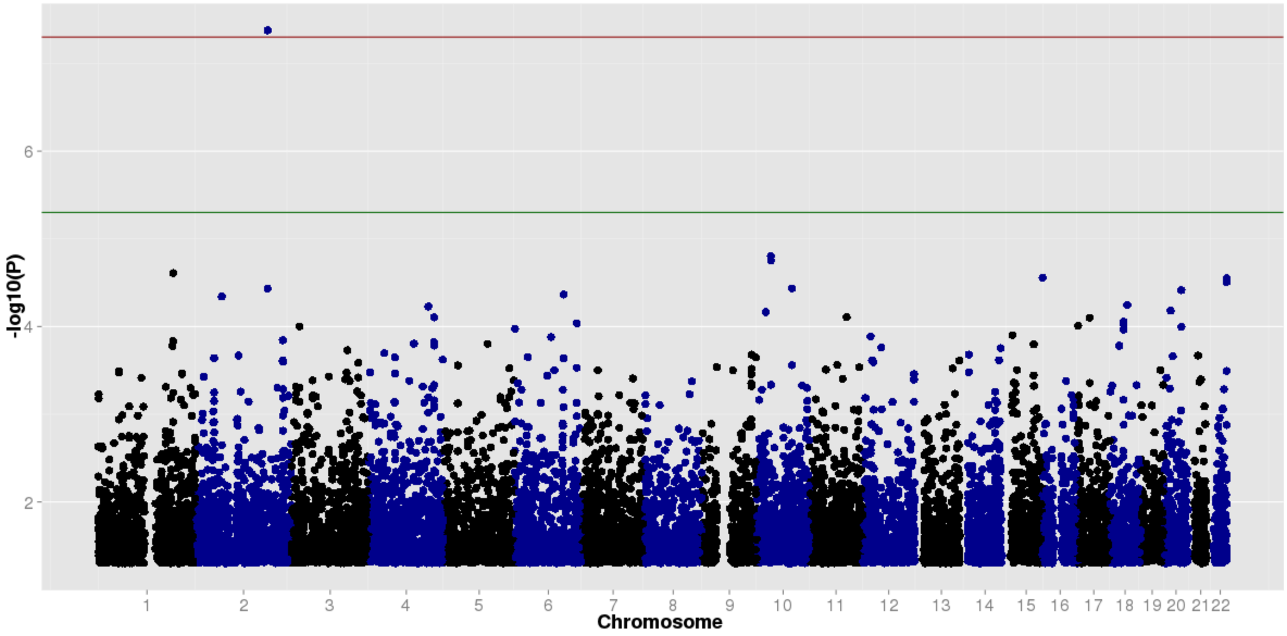

Figure S6

Manhattan plot for GWAS analyses of case sex restricted to genotyped markers in the iPSYCH sample. The horizontal red line indicates genome-wide significance (p<5E-8) and the horizontal green line indicates suggestive sub-threshold signals (p<5E-6).

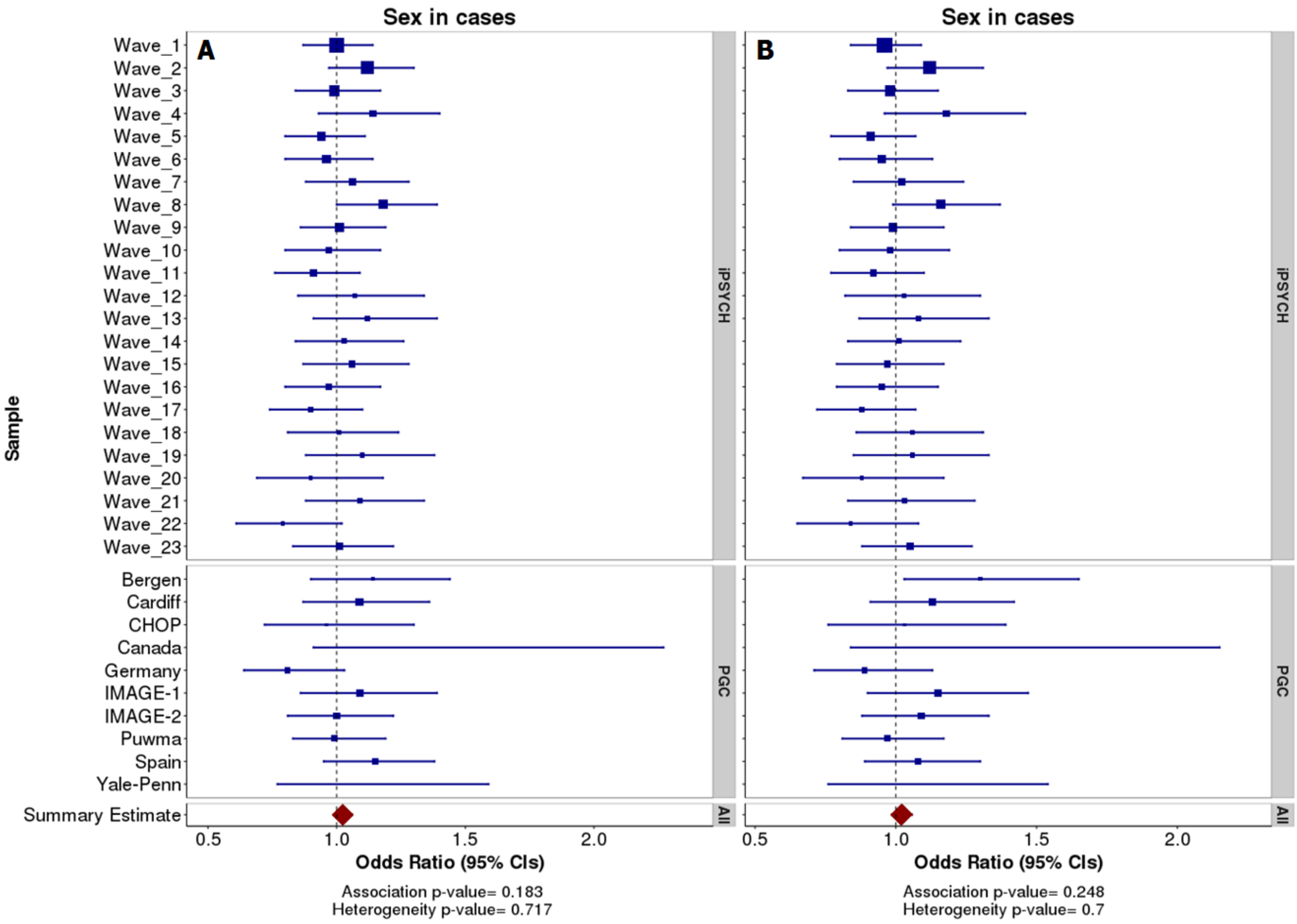

Figure S7

Forest plots of meta-analysis results for logistic regression analyses of ADHD polygenic risk score with case sex as the outcome. Sensitivity analyses exclude 23andMe and non-European ancestry individuals from the GWAS discovery sample. Polygenic risk scores are based on GWAS: a) with and b) without sex as a covariate.

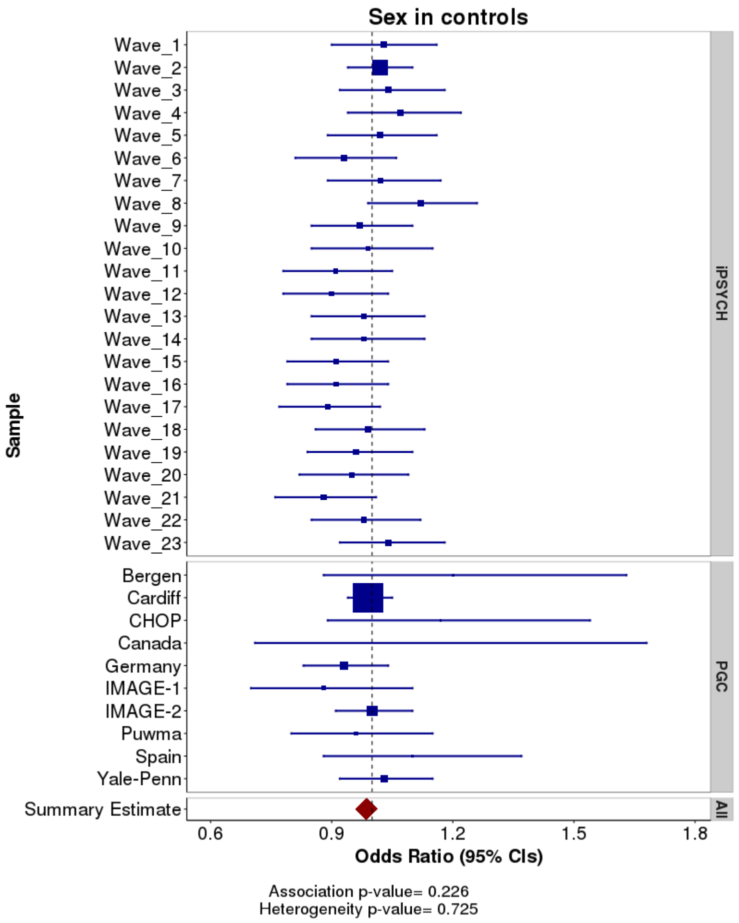

Figure S8

Forest plot of meta-analysis results for logistic regression analyses of ADHD polygenic risk score with control sex as the outcome.

Supplemental References

1. Demontis D, Walters RK, Martin J, Mattheisen M, Als TD, Agerbo E, et al. Discovery Of The First Genome-Wide Significant Risk Loci For ADHD. bioRxiv. 2017;

2. Neale BM, Lasky-Su J, Anney R, Franke B, Zhou K, Maller J, et al. Genome-wide association scan of attention deficit hyperactivity disorder. Am J Med Genet (Neuropsychiatric Genet. 2008;147B(8):1337–44.

3. Neale BM, Medland SE, Ripke S, Asherson P, Franke B, Lesch K-PP, et al. Meta-analysis of genome-wide association studies of attention-deficit/hyperactivity disorder. J Am Acad Child Adolesc Psychiatry. Elsevier Inc.; 2010 Sep;49(9):884–97.

4. Mick E, Todorov A, Smalley S, Hu X, Loo S, Todd RD, et al. Family-based genome-wide association scan of attention-deficit/hyperactivity disorder. J Am Acad Child Adolesc Psychiatry. Elsevier; 2010 Sep;49(9):898–905.e3.

5. Elia J, Gai X, Xie HM, Perin JC, Geiger E, Glessner JT, et al. Rare structural variants found in attention-deficit hyperactivity disorder are preferentially associated with neurodevelopmental genes. Mol Psychiatry. 2009/06/24. 2010;15(6):637–46.

6. Lionel AC, Crosbie J, Barbosa N, Goodale T, Thiruvahindrapuram B, Rickaby J, et al. Rare Copy Number Variation Discovery and Cross-Disorder Comparisons Identify Risk Genes for ADHD. Sci Transl Med. 2011;3(95):95ra75.

7. Neale BM, Medland S, Ripke S, Anney RJL, Asherson P, Buitelaar J, et al. Case-control genome-wide association study of attention-deficit/hyperactivity disorder. J Am Acad Child Adolesc Psychiatry. 2010/08/25. Elsevier Inc.; 2010 Sep;49(9):906–20.

8. Stergiakouli E, Hamshere M, Holmans P, Langley K, Zaharieva I, Hawi Z, et al. Investigating the contribution of common genetic variants to the risk and pathogenesis of ADHD. Am J Psychiatry. Am Neuropsych Assoc; 2012 Feb;169(2):186–94.

9. Sánchez-Mora C, Ribasés M, Ramos-Quiroga JA, Casas M, Bosch R, Boreatti-Hümmer A, et al. Meta-analysis of brain-derived neurotrophic factor p.Val66Met in adult ADHD in four European populations. Am J Med Genet B Neuropsychiatr Genet. 2010 Mar 5;153B(2):512–23.

10. Hinney A, Scherag A, Jarick I, Albayrak Ö, Pütter C, Pechlivanis S, et al. Genome-wide association study in German patients with attention deficit/hyperactivity disorder. Am J Med Genet Part B Neuropsychiatr Genet. 2011 Dec;156B(8):888–97.

11. Yang L, Neale BM, Liu L, Lee SH, Wray NR, Ji N, et al. Polygenic transmission and complex neuro developmental network for attention deficit hyperactivity disorder: Genome-wide association study of both common and rare variants. Am J Med Genet Part B Neuropsychiatr Genet. 2013;162B(5):419–30.

12. Zayats T, Johansson S, Haavik J. Expanding the toolbox of ADHD genetics. How can we make sense of parent of origin effects in ADHD and related behavioral phenotypes? Behav Brain Funct. BioMed Central; 2015 Jan 16;11(1):33.

13. Gelernter J, Kranzler HR, Sherva R, Almasy L, Koesterer R, Smith AH, et al. Genome-wide association study of alcohol dependence:significant findings in African- and European-Americans including novel risk loci. Mol Psychiatry. 2014 Jan;19(1):41–9.

14. Gelernter J, Sherva R, Koesterer R, Almasy L, Zhao H, Kranzler HR, et al. Genome-wide association study of cocaine dependence and related traits: FAM53B identified as a risk gene. Mol Psychiatry. 2014 Jun;19(6):717–23.

15. Gelernter J, Kranzler HR, Sherva R, Koesterer R, Almasy L, Zhao H, et al. Genome-wide association study of opioid dependence: multiple associations mapped to calcium and potassium pathways. Biol Psychiatry. 2014 Jul 1;76(1):66–74.

16. Sherva R, Wang Q, Kranzler H, Zhao H, Koesterer R, Herman A, et al. Genome-wide Association Study of Cannabis Dependence Severity, Novel Risk Variants, and Shared Genetic Risks. JAMA psychiatry. 2016 Mar 30;

17. Bulik-Sullivan B, Finucane HK, Anttila V, Gusev A, Day FR, Loh P-R, et al. An atlas of genetic correlations across human diseases and traits. Nat Genet. Nature Research; 2015 Sep 28;47(11):1236–41.

18. Bulik-Sullivan B, Loh P-R, Finucane HK, Ripke S, Yang J, Patterson N, et al. LD Score regression distinguishes confounding from polygenicity in genome-wide association studies. Nat Genet. Nature Research; 2015 Feb 2;47(3):291–5.

19. Yang J, Lee SH, Goddard ME, Visscher PM. GCTA: a tool for genome-wide complex trait analysis. Am J Hum Genet. 2011 Jan 7;88(1):76–82.

20. Lee SH, Yang J, Goddard ME, Visscher PM, Wray NR. Estimation of pleiotropy between complex diseases using single-nucleotide polymorphism-derived genomic relationships and restricted maximum likelihood. Bioinformatics. Oxford University Press; 2012 Oct 1;28(19):2540–2.

21. Polanczyk G, de Lima MS, Horta BL, Biederman J, Rohde LA. The worldwide prevalence of ADHD: a systematic review and metaregression analysis. Am J Psychiatry. Am Psychiatric Assoc; 2007 Jun;164(6):942.

22. The International Schizophrenia Consortium. Common polygenic variation contributes to risk of schizophrenia and bipolar disorder. Nature. 2009;460(7256):748–52.

23. Wray NR, Lee SH, Mehta D, Vinkhuyzen AAE, Dudbridge F, Middeldorp CM. Research Review: Polygenic methods and their application to psychiatric traits. J Child Psychol Psychiatry. 2014 Aug 1;55(10):1068–87.

24. Ludvigsson JF, Otterblad-Olausson P, Pettersson BU, Ekbom A. The Swedish personal identity number: possibilities and pitfalls in healthcare and medical research. Eur J Epidemiol. Springer Netherlands; 2009 Nov 6;24(11):659–67.

25. Ludvigsson JF, Almqvist C, Bonamy A-KE, Ljung R, Michaëlsson K, Neovius M, et al. Registers of the Swedish total population and their use in medical research. Eur J Epidemiol. Springer Netherlands; 2016 Feb 14;31(2):125–36.

26. Ekbom A. The Swedish Multi-generation Register. In 2011. p. 215–20.

27. Ludvigsson JF, Andersson E, Ekbom A, Feychting M, Kim J-L, Reuterwall C, et al. External review and validation of the Swedish national inpatient register. BMC Public Health. BioMed Central; 2011 Dec 9;11(1):450.

28. Wettermark B, Hammar N, MichaelFored C, Leimanis A, Otterblad Olausson P, Bergman U, et al. The new Swedish Prescribed Drug Register—Opportunities for pharmacoepidemiological research and experience from the first six months. Pharmacoepidemiol Drug Saf. John Wiley & Sons, Ltd.; 2007 Jul;16(7):726–35.
